# Low-cost production and application of lipopeptide for bioremediation and plant growth by *Bacillus subtilis* SNW3

**DOI:** 10.1186/s13568-021-01327-0

**Published:** 2021-12-11

**Authors:** Aiman Umar, Aneeqa Zafar, Hasina Wali, Meh Para Siddique, Muneer Ahmed Qazi, Afshan Hina Naeem, Zulfiqar Ali Malik, Safia Ahmed

**Affiliations:** 1 Department of Microbiology, Faculty of Biological Sciences, Quaid-i-Azam University, Islamabad, 45320 Pakistan; 2 Department of Microbiology, University of Balochistan, Quetta, 87300 Pakistan; 3 Department of Microbiology, Shah Abdul Latif University, Khairpur, Sindh 66111 Pakistan; 4 Department of Psychology, Quaid-i-Azam University, Islamabad, 45320 Pakistan

**Keywords:** Ecotoxicity, *Bacillus subtilis* SNW3, Lipopeptide, Bioremediation, Plant growth promotion

## Abstract

At present time, every nation is absolutely concern about increasing agricultural production and bioremediation of petroleum-contaminated soil. Hence, with this intention in the current study potent natural surfactants characterized as lipopeptides were evaluated for low-cost production by *Bacillus subtilis* SNW3, previously isolated from the Fimkessar oil field, Chakwal Pakistan. The significant results were obtained by using substrates in combination (white beans powder (6% w/v) + waste frying oil (1.5% w/v) and (0.1% w/v) urea) with lipopeptides yield of about 1.17 g/L contributing 99% reduction in cost required for medium preparation. To the best of our knowledge, no single report is presently describing lipopeptide production by *Bacillus subtilis* using white beans powder as a culture medium. Additionally, produced lipopeptides display great physicochemical properties of surface tension reduction value (SFT = 28.8 mN/m), significant oil displacement activity (ODA = 4.9 cm), excessive emulsification ability (E24 = 69.8%), and attains critical micelle concentration (CMC) value at 0.58 mg/mL. Furthermore, biosurfactants produced exhibit excellent stability over an extensive range of pH (1–11), salinity (1–8%), temperature (20–121°C), and even after autoclaving. Subsequently, produced lipopeptides are proved suitable for bioremediation of crude oil (86%) and as potent plant growth-promoting agent that significantly (P < 0.05) increase seed germination and plant growth promotion of chili pepper, lettuce, tomato, and pea maximum at a concentration of (0.7 g/100 mL), showed as a potential agent for agriculture and bioremediation processes by lowering economic and environmental stress.

**Supplementary Information:**

The online version contains supplementary material available at 10.1186/s13568-021-01327-0.

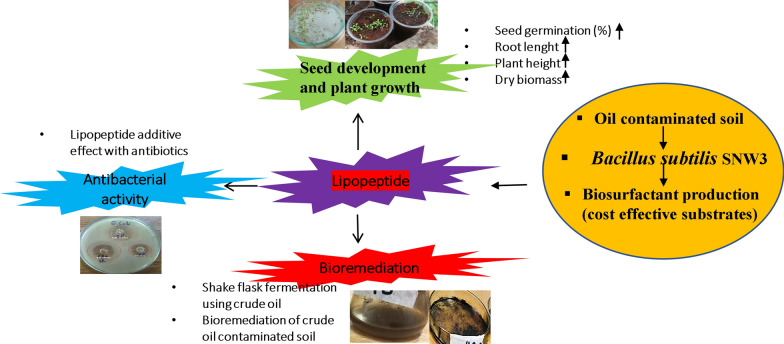

## Introduction

Environmental pollution due to petroleum products such as crude oil, diesel, and gasoline is of major ecological concern nowadays (Jimoh and Lin 2019). Major health problems in humans and animals are occurred due to the release of petroleum and its by-products in a terrestrial and aquatic ecosystem because of having mutagenic, carcinogenic, and teratogenic effects (Yadav et al. 2016). Petroleum-derived pollutants result in the limitation of phosphorus, iron, and nitrogen availability in agricultural soil (Nogueira et al. 2011). In today’s challenging world enhanced agricultural productivity is the need of the hour to encounter human food demands. However, equally alarming is the damage of agricultural land by pollutants that needs bioremediation strategies. Hence, researchers must focus on remediation of all these issues. Biosurfactants are amphiphilic secondary metabolites that exhibit surface-active properties produced by bacteria, fungi, and yeast (Santos et al. 2016). Biosurfactant-producing microorganisms enhance plant growth through improvement in plant immunity against organic contaminants in the environment. Furthermore, they are also efficient in alleviating stress responses in plants along with strengthening plant growth and development (Almansoory et al. 2019). The surfactin preferably and to the lower extent fengycin, lipopeptides are capable to provoke defense responses that generate signaling molecules for activation of induced systemic resistance (ISR) in plants (Ongena et al. 2007). Other lipopeptides that are reported for induction of plant defense response includes iturin (Yamamoto et al. 2015), mycosubtilin (Farace et al. 2015), bacillomycin D (Wu et al. 2018), and sessilin and orfamide (D'aes et al. 2014). One of the positive influences of the use of lipopeptides in agriculture is its biocompatibility with living organisms (Ławniczak et al. 2013). Hence, to minimize the initial dose of fertilizers by seed stimulation strategies and its equal distribution in the soil is made possible through biosurfactants (Krawczyńska et al. 2012). Many researchers verified that plant growth-promoting rhizobacteria (PGPR) positively enhance plant development after association with the hydrocarbon-degrading bacteria in the contaminated soil (Pawlik et al. 2017). Different plant growth-promoting traits include phosphate solubilization, siderophore production, hydrogen cyanide (HCN) production, indole acetic acid (IAA) production, and systemic resistance induction (Benaissa 2019). Hence, for employing biosurfactants in agriculture, bioremediation and its application in other fields the reduction in cost needed for production are of absolute concern (Jimoh and Lin 2019). Increase in awareness among public about the use of environment-friendly and sustainable green products demand new strategies development to cut down the production cost for replacement of toxic synthetic surfactants with biosurfactants (Shaban and Abd-Elaal 2017). Biosurfactants with numerous useful applications provide growing interest in diverse industrial sectors including food, medicine, cosmetics, and agriculture (Patil et al. 2014). However, the production cost is still high that depends on the availability of raw materials and downstream processing for scaleup at the industrial level (Akbari et al. 2018). Raw materials used for biosurfactant production accounts for about 50% of the final production cost (Rufino et al. 2007). Better choice of raw material is a way to cut down the budget and make the process economically feasible (Jimoh and Lin 2019; Mukherjee et al. 2006). Unlike synthetic surfactants that are produced from petroleum feed stock, biosurfactants could be produced using waste materials like agriculture waste (wheat bran), brewery waste, and food waste by-products (potato peels and waste frying oil) that not only reduce cost but also helps in waste disposal in environment-friendly manner (Moshtagh et al. 2018; Vea et al. 2018). In the present study, we used potato peels powder, waste frying oil, molasses, and white beans powder as a low-cost substrate for biosurfactant production. Hence, with all the above intentions the current study was conducted to produce stable potent biosurfactants employing various cost-effective renewable resources and to evaluate the potential of produced lipopeptides for detoxification and management of crude oil contaminated soil and to promote plant growth and development.

## Materials and methods

### Materials and chemicals

All chemicals used in the study werepurchased from Sigma-Aldrich (Merck KGaA, Darmstadt, Germany), and are of analytical grade. The standard surfactin (≥ 98% purity) used as a reference for lipopeptides characterization in this study was obtained from Sigma-Aldrich. Fertilizers (NPK; 20-10-10) used in the study were bought from Agro-chemicals, Fertilizers (TAK Agro Brand). Antibiotics used in the current study were purchased from Werrick Pharmaceuticals Pakistan. Crude oil used was collected from Pakistan petroleum limited.

### Microorganism and culture conditions

In the current study, *Bacillus subtilis* SNW3 (Genbank Acc. No. JX534509.1), obtained from Microbiology Research Lab, Quaid-i-Azam University, Islamabad, was previously identified and isolated from contaminated soil of Fimkessar oil field, Chakwal, Pakistan (Malik and Ahmed 2012). This strain SNW3 also referred to as QVS1 is deposited with the Belgian Coordinated Collections of Microorganisms BCCM/LMG, Ghent, Belgium, under Accession Number “LMG P-30406”. The bacterial sample was cultured on nutrient agar plates (Yeast extract 2.0; Beef extract 1.0; Peptone 5.0; Sodium chloride 5.0; Agar 15 g/L) incubated for 24 h at 30°C to obtain separate pure colonies, stored for regular use at 4°C and sub-cultured before use. The strain was preserved at − 80°C in nutrient broth (Peptone, 5; Meat extract, 1; Yeast extract, 2.0 and sodium chloride g/L) supplemented with 30% glycerol.

### Cost-effective substrates for biosurfactant production

For low cost biosurfactant production various cost-effective substrates were evaluated that includes: potato peels powder (total carbohydrate 68.7%; starch 25%; protein 18%; non-starch polysaccharide 30%; acid-soluble and acid-insoluble lignin 20% and nitrogen 1.3%) (Liang et al. 2014), molasses (total sugars 62.3%, sucrose 48.8%, starch 0.33% and ash 13.1%) (Palmonari et al. 2020), white beans powder (protein 15.62%; carbohydrates 60.47%; lipids 2.13%; crude fibre 14.15%) (Alayande et al. 2012), waste frying oil (palmitic acid 15.86%; oleic acid 29.83%; stearic acid 4.87% and linoleic acid 28.85%) (Banani et al. 2015) and nitrogen sources: sodium nitrite, urea and ammonium nitrate while, conventional media yeast extract (protein 62.5%; sugar 2.90 %; fat 0.10 %; ash 9.50 %) was used as control. Each carbon source listed above was designed to use individually, then selected substrates were used in different combinations to achieve an optimized medium composition. Molasses used in current study was obtained from Chashma Sugar Mills Limited in Dera Ismail Khan (Pakistan). Potato peels and waste frying oil were obtained from café located at Quaid-i-Azam University Islamabad (Pakistan). Whereas white beans were obtained from National Agricultural Research Council (NARC) Islamabad Pakistan.

Cost-effective substrates were categorized through various methods: soluble total organic nitrogen analysis through the Kjeldahl method (Toledo et al. 2018), soluble total organic carbon with a TOC analyzer (Multi N/C 3100, Analytic Jena), and Dumas method for total organic carbon (TOC) and total organic nitrogen in the solid fraction by applying (LECO, TruSpec CHN) tool (Munera-Echeverri et al. 2020).

### Inoculum

*Bacillus subtilis* SNW3, streaked and stored on nutrient agar plates at 4°C was used for inoculum preparation. A loop full of culture from a single isolated colony on plate added in 100 mL nutrient broth (Peptone, 5; Meat extract, 1; Yeast extract, 2.0 and sodium chloride, 5 g/L) incubated at 30°C for 48 h then seed culture from the nutrient broth was used as inoculum for all experiments.

### Production optimization, extraction, and partial purification of biosurfactant

The strain *Bacillus subtilis* SNW3 was grown on conventional yeast extract media (2% w/v) and mineral salt medium (MSM) as described by (Abouseoud et al. 2008; Rastogi et al. 2021) of given composition (g/L: KH_2_PO_4_, 2.0; K_2_HPO_4_, 4.0; FeSO_4_·7H_2_O, 0.025; MgSO_4_·7H_2_O, 1.0; KCl, 0.2; NaCl, 5.0; CaCl_2_·2H_2_O, 0.02; and trace elements solution with composition of MnSO_4_·4H_2_O, 1.78; ZnSO_4_·7H_2_O, 2.32; CuSO_4_·5H_2_O, 1.0; H_3_BO_3_, 0.56; KI, 0.66 and NH_4_MoO_4_·2H_2_O, 0.39). Different environmental process parameters significant for biosurfactant production were evaluated using above mentioned media at various range of temperature (15, 30, 37 and 50°C), pH (2, 4, 6, 8, 10, 12), agitation speed (0, 150 and 250 rpm) and inoculum size (0.5, 1, 1.5, 2 and 2.5). Initially, three nitrogen sources (urea, sodium nitrate, and ammonium nitrate) and four cost-effective substrates (white beans powder, potato peels powder, waste frying oil, and molasses) were tested separately. After that for different combinations, the selected nitrogen source and cost-effective substrates added with MSM were used in various combined media compositions. Optimization of substrate and culture conditions are given in (Table 1). Yeast extract as the most preferable substrate for biosurfactant production was used as control media (Qazi et al. 2013). The designed experiments for substrate evaluation were run with 100 mL media in 250 mL Erlenmeyer flask with pH adjusted to 7.0 ± 0.2 and kept in a shaker for 96 h of incubation at 30°C and 150 rpm. The cell-free supernatant obtained after centrifugation at 12,000 rpm was acidified up to pH 2.0 with 1 M hydrochloric acid (HCL) and kept overnight at 4 °C. For bacterial biomass production, the collected pallet was washed with saline solution (0.9% w/v NaCl), oven-dried at 100°C, and weighted (Guerfali et al. 2020).

**Table 1:** Optimization of low-cost substrate and culture conditions for lipopeptide production by *Bacillus subtilis* SNW3

Factors	Ranges
Temperature (°C)	15, 30, 37 and 50
pH	2, 4, 6, 8, 10, 12
Agitation speed (rpm)	0, 150, 250
Inoculum size (%, w/v)	0.5, 1, 1.5, 2, 2.5
Nitrogen source (0.1%, w/v)	Urea, sodium nitrate, ammonium nitrate
Carbon source (conc. 2%, w/v)	White beans powder, potato peels powder, molasses, waste frying oil, yeast extract
Carbon and nitrogen source ratio (W.B.P + W.F.O + U) w/v	11 + 0.5 + 0.1, 8 + 1 + 0.1, 6 + 1.5 + 0.1, 3 + 2 + 0.1

### Dry weight of lipopeptides

For crude biosurfactants, pelleted precipitates were extracted with chloroform/methanol (2:1) and concentrated by rotary evaporation (Marchut-Mikolajczyk et al. 2018). After that concentrate was poured into a pre-weighted sterile beaker. The crude lipopeptides were oven-dried at 60 °C for 24 h. Plates were weighted after drying (Anandaraj and Thivakaran 2010). The following formula was used to calculate the dry weight of crude lipopeptide extract produced by *Bacillus subtilis* SNW3 under optimized substrate and culture conditions. Dry weight of lipopeptide produced = (Weight of plate containing dried lipopeptide − Empty plate weight).

### Assessment of biosurfactant production

For quantitative analysis of biosurfactant production, various assays were used that include surface tension measurement (SFT), oil displacement assay (ODA), and emulsification index (E24). The sample used for analysis was in the form of cell-free supernatant (CFS).

### Oil displacement activity (ODA)

For estimation of biosurfactant production oil displacement activity (ODA) was performed according to the method of Yalçın et al. (2018). Briefly, 20 µL of crude oil was add on the surface of 40 mL distilled water in the petri dish. The cell-free supernatant (CFS) of 10 µL was placed gently on a uniform crude oil layer formed on distilled water. Oil layer was displaced, and clear zone diameter was measured in centimetre (cm). Production medium without inoculum was used as negative control. Clear zone formation indicates biosurfactant presence in CFS.

### Emulsification index (E24)

Emulsification index (E24%) was used to estimate the emulsifying capacity of the biosurfactant, performed through protocol of Ferhat et al. (2011) with minor modifications. In short, kerosene oil (2 mL) and an equal volume of cell-free supernatants were added in the test tube and mixed for 2 min on the vertex mixer. After that, these test tubes were kept undisturbed for 24 h at room temperature. The percent emulsification was measured using given formula where heights were calculated in centimetres (cm).

$${\text{E}}_{24} \left( \% \right) = \frac{{{\text{Height}}\;{\text{of}}\;{\text{the}}\;{\text{emulsion}} \left( {{\text{cm}}} \right)}}{{{\text{Total}}\;{\text{height}}\;{\text{of}}\;{\text{the}}\;{\text{solution}} \left( {{\text{cm}}} \right)}} \times 100.$$

### Surface tension (SFT) measurement

For quantitative analysis of biosurfactant produced by *Bacillus subtilis* SNW3 surface tension (SFT) of the cell-free supernatant was measured in mN/m by using KRUSS K20 digital Tensiometer (Kruss GmbH, Hamburg, Germany). SFT measurement was performed at room temperature by Wilhelmy plate through according to the protocol given by the manufacturer. Cell-free supernatant of 25 to 30 mL was put on the tensiometer platform in a glass cup. Wilhelmy plate was sterilized before use, adjusted on the tensiometer, submerged in the broth followed by surface tension measurement (Novikov et al. 2017).

### Structural characterization of lipopeptide by thin-layer chromatography (TLC) and Fourier transform infrared spectroscopy (FTIR)

For thin-layer chromatography (TLC) and Fourier transform infrared spectroscopy (FTIR) analysis, extracted form of crude biosurfactant was used while surfactin a class of lipopeptide (from sigma) was taken as standard for initial characterization. Crude biosurfactant components were separated on Silica coated aluminum plates, silica gel 60 F254, MERCK Germany using chloroform: methanol: acetic acid (85:10:5, v/v) visualized under the wavelength of 254 and 365 nm to find retention factor (Rf) (Joy et al. 2017). For determination of chemical nature of bonds and, functional groups present in the crude form of biosurfactant produced FTIR analysis was performed. 10 mg of crude biosurfactant was loaded and the spectrum was observed at the range of 4500–450 cm^−1^ using Tensor 27 (Bruker) FTIR spectrophotometer, equipped with ZnSe ATR (Marchut-Mikołajczyk et al. 2019).

### Determination of critical micelles concentration (CMC) and critical micelle dilution (CMD) of lipopeptides

The CMC of the produced biosurfactant was analyzed through change in surface tension reduction values with varying concentrations of 0.06 to 1.24 mg/mL prepared in demineralized water (Datta et al. 2018). For critical micelle dilution cell, free supernatant was diluted10-folds up to three levels (i.e. 10×, 100×, and 1000×) named as CMD^−1^, CMD^−2^, and CMD^−3^, respectively. Surface tension reduction value was analyzed by Wilhelmy plate method using KRUSS K20 digital Tensiometer (Kruss GmbH, Hamburg, Germany), performed at room temperature (Campos et al. 2019).

### Lipopeptides stability studies

To elucidate the thermal stability of lipopeptide, thestandard solutions of crude biosurfactant were prepared at a concentration of 600 mg/L and incubated at different temperatures (20–121 °C) for 1 h. Furthermore, a stability test of the produced lipopeptide at saline conditions was performed. Different concentrations of sodium chloride NaCl (1–10%) were added to the biosurfactant solutions and incubated at 30 °C for 1 h. To determine pH effect on lipopeptide activity different buffer solutions were prepared and added to the biosurfactant standard solution, adjusted to pH 1–5 using citrate–phosphate buffer, pH 7 using phosphate buffer, and pH 9–11 using carbonate-bicarbonate buffer solutions, incubated for 30 min. The stability of the produced lipopeptide was checked through surface tension reduction value of each treated sample (Goswami and Deka 2019).

### Functional characterization of the lipopeptides

#### Lipopeptide screening for antimicrobial activity

Lipopeptide for its antimicrobial potential was assessed through well diffusion assay as mentioned before Singh et al. (2014). Mueller–Hinton agar plates were prepared to contain *Escherichia coli* ATCC 25922 and *Salmonella typhi* ATCC 14028. The crude lipopeptide (10 mg/mL), ciprofloxacin and clarithromycin (1 mg/mL), and biosurfactant in addition with antibiotics (5:0.5 mg/mL) were poured at a concentration of 100 µL and kept at 37°C for 24 h of incubation. To determine the bacterial sensitivity to lipopeptide, the diameter of inhibition zone (mm) was measured according to Clinical Laboratory Standards Institute (Wayne 2002). To investigate the additive effect of lipopeptide with antibiotics any increase in the diameter of the inhibition zone was measured as compared to antibiotics. Antibiotics without biosurfactants were used as a positive control (Ekprasert et al. 2020).

#### Exploration of lipopeptides for seeds germination and plant growth

The seeds of tomato (*Solanum Lycopersicum*), pea (*Pisum sativum*), chili pepper (*Capsicum annuum*), and lettuce (*Lactuca sativa*) were collected from National Agricultural Research Centre (NARC) Islamabad, Pakistan. Obtained seeds were surface sterilized with 10% Na–hypochlorite for 20 min and then washed with sterile distilled water before use. The crude lipopeptide extract produced with cost-effective optimized media was used in this assay. The seed germination experiment was conducted in a petri plate containing 40 seeds positioned in filter paper and cotton soaked with four different concentrations (0.1, 0.3, 0.5, and 0.7 g/100 mL) of crude lipopeptide solution. Distilled water100% v/v was used as a control. These plates were kept in yellow light at 25 °C for 7 days after that relative seed germination (G, %): [No. of seeds germinated (treatment)/No. of seeds germinated (control) × 100] was calculated. Following the germination test seeds treated with lipopeptide solution were transferred in pots (seeds without pre-treatment with biosurfactant were used as control) and kept in a greenhouse with temperature maintained between 20 and 22 °C. Furthermore, for plant growth stimulation crude lipopeptide solution was added in pots at a concentration (0.1, 0.3, 0.5, and 0.7 g/100 mL) dissolved in distilled water thrice with 10 days interval while in control pots pure water was added. The emergence of plant seedlings was tested and checked for the morphological characteristic of plants like shoot length (mm), root length (mm), and dry weight (g) of plants after 40 days (Huang et al. 2017).

#### Bioremediation assay in shake flask fermentation

The biodegradation efficiency of crude oil by *Bacillus subtilis* SNW3 was analyzed as illustrated by Varjani and Upasani (2016) with minor changes. An aliquot of 2 mL pre cultured *Bacillus subtilis* SNW3 was transferred into 250 mL of Erlenmeyer flask containing 100 mL mineral salt media and different concentrations 0.5, 1, 1.5, and 2% (v/v) of crude oil. For monitoring of abiotic loss of the crude oil, an uninoculated media was used as control. All these flasks were incubated for 21 days at 200 rpm and 35C. The bacterial growth in crude oil was detected through measurement of the absorbance at (OD600 nm) through spectrophotometer while SFT was measured by tensiometer. To estimate the residual crude oil in media, crude oil was extracted with hexane, left for evaporation in a pre-weight clean beaker. For quantification of remaining crude oil after degradation gravimetric analysis was performed at different time intervals by following the formula proposed by Patowary et al. (2017).

$${\text{Hydrocarbon}}\;{\text{degradation }}\% = {\text{Amount}}\;{\text{of}}\;{\text{crude}}\;{\text{oil}}\;{\text{degraded}}/{\text{Amount}}\;{\text{of}}\;{\text{crude}}\;{\text{oil}}\;{\text{added}}\;{\text{in}}\;{\text{the}}\;{\text{media}} \times 100.$$

### Bioremediation of crude oil in the soil through various design treatments

In this assay bioremediation of crude oil contaminated soil was monitored by collecting garden soil from Quaid-i-Azam University Islamabad. Biosurfactant suitability for removing hydrophobic pollutants from soil was analyzed by collecting 5–10 cm deep topsoil while following the protocol of Okop et al. (2012) and transported in a clean container to the Microbiology laboratory of Quaid-i-Azam University Islamabad Pakistan. The soil collected was air dried and sieved with a 2 mm sieve after that 5% of crude oil was sprayed on the soil to pollute soil homogenically. The polluted soil was left undisturbed for 5 days and then divided into 200 g of equal parts and dispensed in pots. These pots were left undisturbed in the open air for a week. Then for conducting bioremediation experiments various designed treatments were established added twice throughout the remediation period: (T0) addition of distilled water as control, (T1) cell-free supernatant obtained after 96 h of incubation with maximum biosurfactant produced by using optimized cost-effective substrate media, (T2) addition of active culture of *Bacillus subtilis* SNW3 (2% inoculum size) cultured in nutrient broth kept in shaker incubator at 30 °C, 150 rpm for 24 h of incubation, (T3) effect of two additives was assessed for bioremediation i.e. T1 + T2, (T4) addition of tween 80 (T5) addition of fertilizer (NPK; 20-10-10) to analyse the effect of fertilizer on bioremediation in comparison to the produced lipopeptide (Pelletier et al. 2004) and (T6) an additional control containing autoclaved soil and 5% crude oil (w/v) was used to examine the crude oil degradation in the soil. Detailed information about all the above treatments is shown in (Table 2). The soil content of each pot was tilled twice a week for aeration with moisture maintenance at 60 % and temperature of 28–30°C, providing all those conditions that are appropriate for crude oil-degrading microbes present in the soil (Agamuthu et al. 2013). After that soil samples of 10 g were collected from different areas of the plastic pots at the 30, 60, 90th day and were gravimetrically determined using the formula given by Ganesh and Lin (2009).

**Table 2:** Different design treatments for removal of crude oil from contaminated soil

Treatments	Soil	Biological treatment	Chemical compounds	Crude oil concentration (%)
Control (T0)	200 g			5
Treatment 1 (T1)	200 g	Cell-free supernatant containing lipopeptide (200 mL)		5
Treatment 2 (T2)	200 g	Active culture of *Bacillus subtilis* SNW3 (100 mL)		5
Treatment 3 (T3)	200 g	T1 (100 mL) + T2 (100 mL)		5
Treatment 4 (T4)	200 g		10 mg/kg of tween 80	5
Treatment 5 (T5)	200 g		0.8 g/kg of fertilizer	5
Treatment 6 (T6)	200 g (autoclaved)			5

### Statistical analysis

The obtained results were analyzed statistically with the use of STATISTICA software, one-way ANOVA (version 8.1). The difference between obtained results was analyzed by using Tukey’s test to find individual and control mean ± standard deviation. Significance value was set at p = 0.05 and p-values ≤ 0.05 were considered significant.

## Results

### Substrate screening and optimization studies for lipopeptide production

**Fig. 1:**
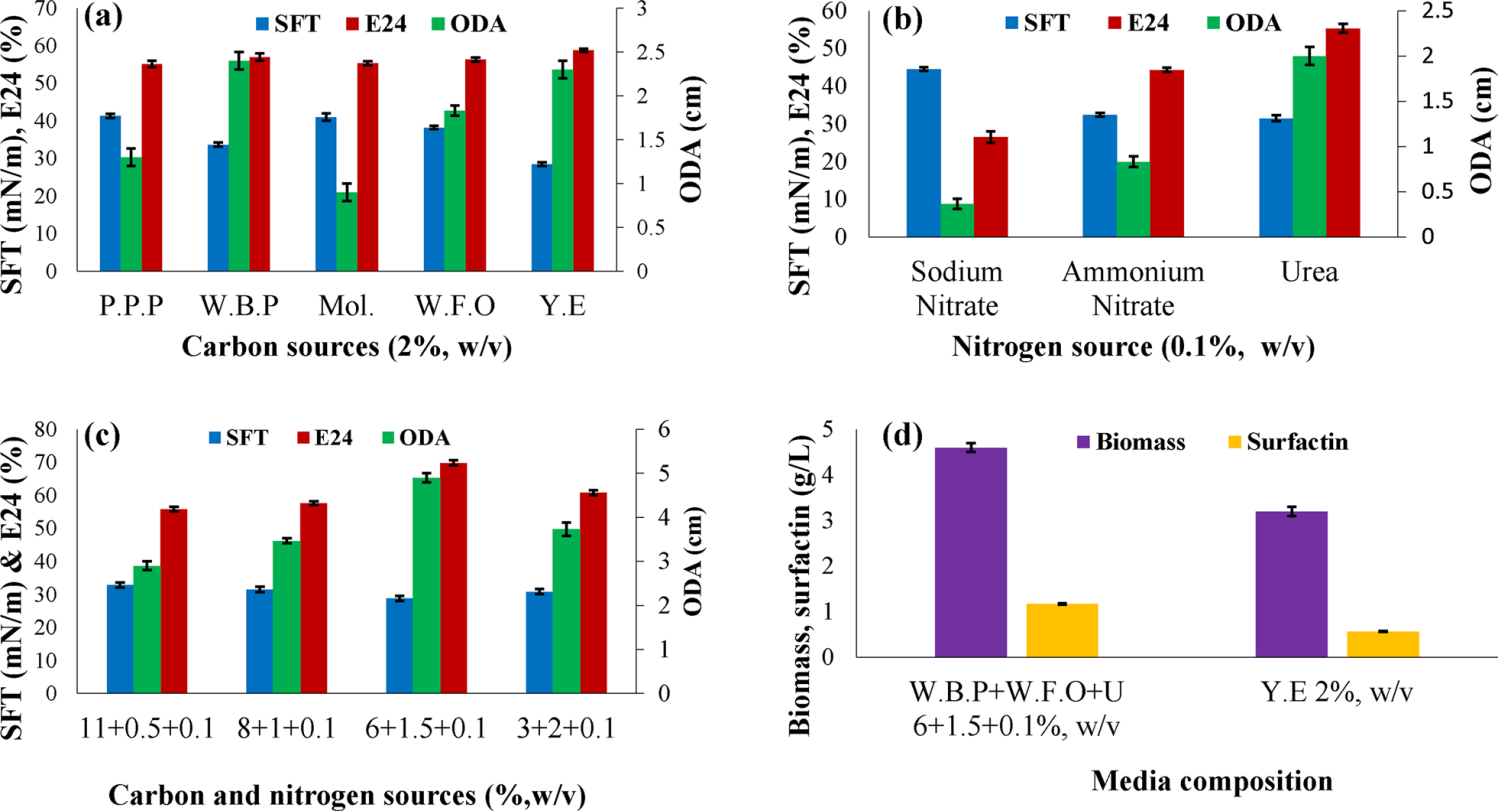
SFT, E24 and ODA values for lipopeptide production by *Bacillus subtilis* SNW3 **a** with alternative carbon sources used individually, **b** different nitrogen sources, **c** with a combination of carbon and nitrogen energy sources and **d** production analysis of surfactin under optimized conditions with yeast extract as a reference, in shake flask fermentation at 30 °C. P.P.P: potato peels powder; W.B.P: white beans powder; Mol.: molasses; W.F.O: waste frying oil; Y.E: yeast extract

Potato peels powder, molasses, white beans powder, and waste frying oil were evaluated as cheap media for lipopeptide production by *Bacillus subtilis* SNW3. Optimized results for culture conditions with 2% yeast extract media showed 30 °C as optimum for maximum lipopeptide production with an ODA value of 1.26 cm While other optimized cultural conditions were with 1% inoculum size, 150 rpm, and pH of 6 (Additional file 1: Fig. S1). At the end of the fermentation process, the obtained ODA values were 1.3, 2.4, 0.9, and 1.8 cm for potato peels powder, white beans powder, sugar cane molasses, and waste frying oil media respectively with 2% w/v concentration. Additionally, the surface tension reduction values observed for all four biosurfactant solutions were reduced from 72 mN/m to 41.3 (potato peels powder), 33.6 (sugar cane molasses), 41 (white beans powder) and 38.2 mN/m (waste frying oil). Though good emulsification values of all these biosurfactant solutions were obtained to about 55 to 57% (Fig. 1a). In the current study among nitrogen sources tested preferably urea act as a good nitrogen source that showed surface tension reduction of 31.4 mN/m and ODA value of 2 cm (Fig. 1b). It was observed that white beans powder and waste frying oil gave significant oil displacement value. the The characterization of substrate samples in terms of total organic carbon and nitrogen content of the yeast extract, white beans powder and potato peels powder used in this study is presented in Additional file 1: Table S1. It has been also found that combination of carbon sources enhances biosurfactant synthesis. The final optimized cost effective media was [white beans powder (6% w/v) + waste frying oil (1.5% w/v) + urea (0.1% w/v)] with significant lipopeptide yield indicated ODA of 4.9 cm, emulsification index of 69.8% and surface tension reduction value up to 28.8 mN/m (Fig. 1c; Additional file 1: Fig. S5a, b).

We aimed the use of alternative non-conventional media for lipopeptide production with optimized media gave 1.17 g/L of crude lipopeptide that was almost double from 0.56 g/L with yeast extract control media, with biomass yield of 4.6 and 3.2 g/L respectively (Fig. 1d). The cost effective usage of media component showed that on average 1 kg of white beans powder with 240 mL of waste frying oil and 640 g of yeast extract media would be enough for preparing 16 L of fermentation media that gave 1.17 g/L of surfactin production.

### Thin layer chromatography analysis

**Fig. 2:**
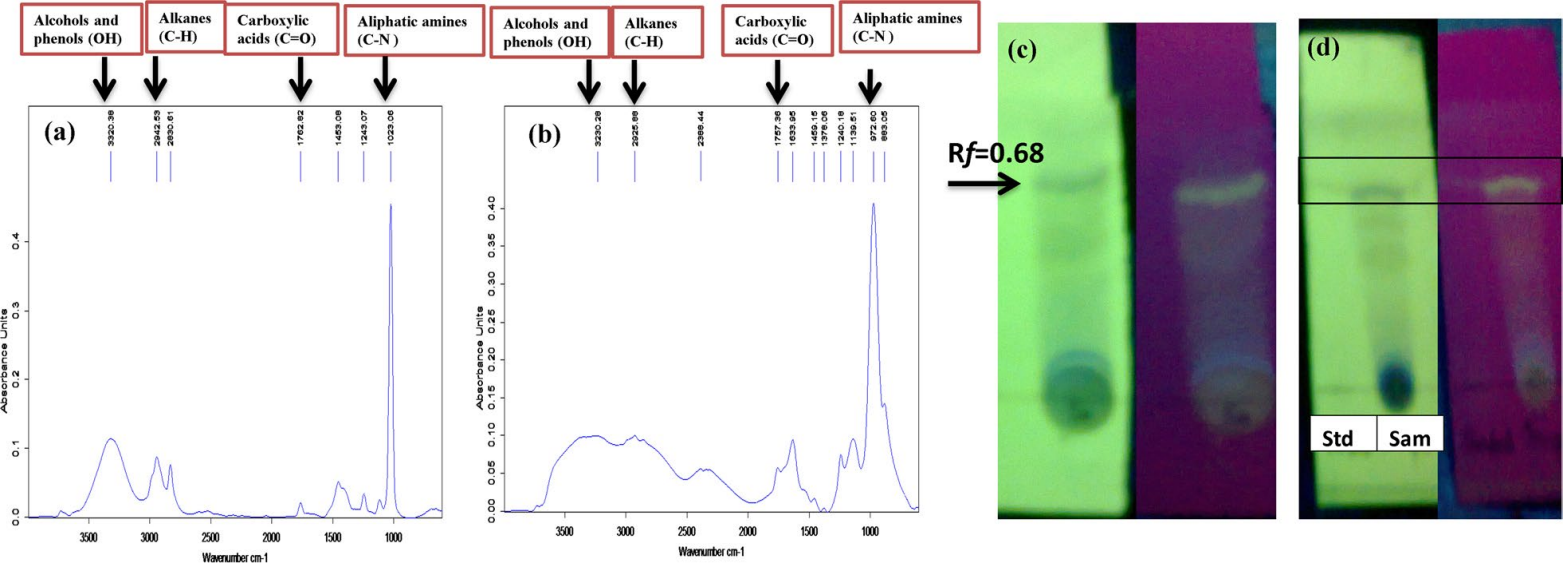
FTIR spectrum and TLC profile of crude lipopeptide produced by *Bacillus subtilis* SNW3 in comparison to standard surfactin show as **a** FTIR of standard surfactin, **b** FTIR of crude lipopeptide extract, **c** TLC profile of crude lipopeptide extract, **d** TLC profile of crude lipopeptide in comparison to standard surfactin

Characterization of crude biosurfactant produced with final optimized media by *Bacillus subtilis* SNW3 was carried out by thin-layer chromatography (TLC) and Fourier transform infrared spectroscopy (FTIR). Results obtained by TLC indicate the lipopeptide nature of the biosurfactant product with most prominentband observed against standard surfactin having retention factor (Rf) value of 0.68 as illustrated in (Fig. 2c, d).

### Structural characterization of biosurfactant produced by FTIR

The FTIR spectra represent the presence of carboxylic functional groups and aliphatic amines the characteristic of the lipopeptide nature of biosurfactant produced. The FTIR spectra show a sharp peak at 1023 cm^−1^ and 972 cm^−1^ that corresponds to the presence of C–N aliphatic amines in standard and crude biosurfactant (Fig. 2a, b). The peaks in FTIR spectra at 1450 and 1130 suggest the presence of stretching bands between carbon atoms and hydroxyl groups. The absorbance appears at 1762 cm^−1^ and 1757 cm^−1^ attributed to the vibrations due to the ester carbonyl group of peptide components. The peaks observed in FTIR spectra at 2942 and 2926 corresponds to the presence of C–H bands (CH_2_–CH_3_ stretching). Another peak ranging from 3500 to 3200 cm^−1^ indicated the presence of alcohols and phenols (O–H stretch, H-bond). The spectra presented in the current study in comparison to standard surfactin from sigma suggested the presence of peptide moiety and aliphatic groups, a distinctive feature of lipopeptides nature of biosurfactant produced by *Bacillus subtilis* SNW3.

### Lipopeptide screening for antimicrobial activity

**Fig. 3:**
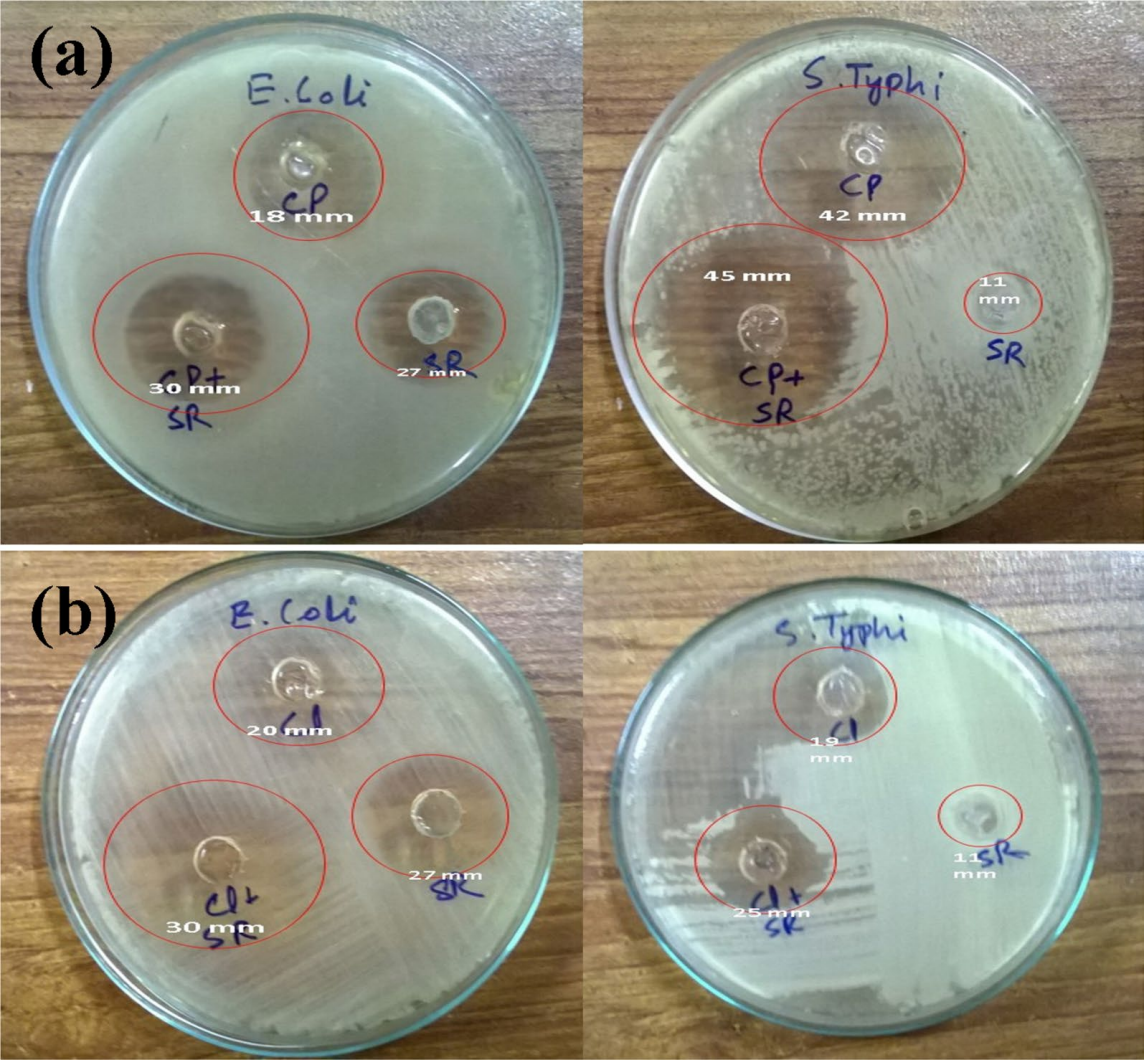
Antibiogram of crude lipopeptide extract tested with antibiotics **a** ciprofloxacin and **b** clarithromycin, against *Escherichia coli* and *Salmonella typhi*

In this study, we observed that lipopeptides produced by *Bacillus subtilis* SNW3 showed antimicrobial and synergistic effects with antibiotics against *Escherichia coli* and* Salmonella typhi*. In the case of lipopeptide alone, the growth of *E. coli* was affected more as compared to *S. typhi.* However, in combination with antibiotics, the results obtained showed an increase in zone of inhibition from 18 to 30 mm for *E. coli* and 42 to 45 mm for *S. typhi* containing lipopeptide plus ciprofloxacin (Fig. 3a). Thus, the addition of lipopeptide renders bacteria more sensitive to ciprofloxacin used in the case of *E. coli*. Similarly, the inhibition zone around lipopeptide plus clarithromycin increased from 20 to 30 mm and 19 to 25 mm for *E. coli* and *S. typhi* (Fig. 3b). In general, lipopeptide showed antibacterial and an additive effect while used in combination with antibiotics, shown in Additional file 1: Fig. S2.

### Critical micelle concentration (CMC) and critical micelle dilution (CMD) determination

The crude biosurfactant obtained from *Bacillus subtilis* SNW3 which was dissolved in distilled water at different concentrations showed a reduction in surface tension of water from 72 to 36 mN/m with an increase in lipopeptide concentration. Lipopeptide produced seems to be more competent exhibited a CMC at 0.5 mg/mL, with surface tension reduction of 36 mN/m (Additional file 1: Fig. S3a). The surface tension values remain stable with an SFT value of 29 mN/m to 32 mN/m after making threefold dilutions showing effective lipopeptide concentration in the medium (Additional file 1: Fig. S3b).

### Stability studies

**Fig. 4:**
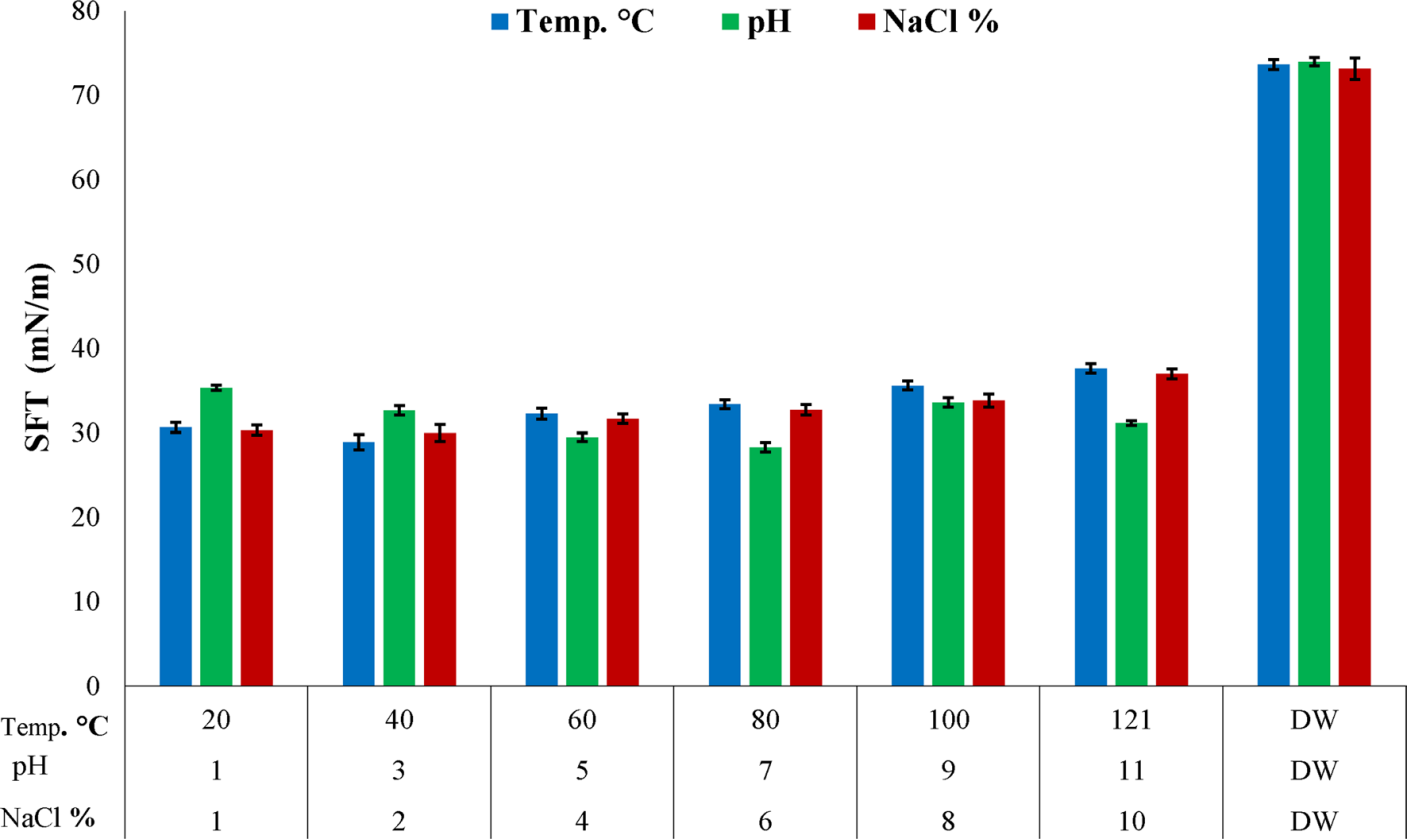
Stability of crude lipopeptide extract against various environmental factors like temperature ranges 20–121 °C, NaCl conc. 1–10% (w/v) and pH ranges 1–11. *DW* distilled water, *Temp* temperature, *NaCl* sodium chloride

The applicability of biosurfactant produced depends on its behavior shows at different conditions of temperature, pH, and salinity (Gudina et al. 2010). The lipopeptide produced during the current study was found to be more stable after exposure to various temperatures ranges since no significant difference was detected for surface tension reduction values from 20 to 121 °C. The favorable surface tension reduction values were observed over a pH range of 1 to 11, although in between pH 5 to 7 lipopeptides produced was found to be more stable (Fig. 4). At pH 1, surface tension value raised slightly up to 35 mN/m that means that produced lipopeptides possess stability at acidic conditions but more effectively stable at alkaline conditions. Besides this, lipopeptide exhibit stablity over a wide range of salt concentrations 1 to 8%, an increase in SFT value at 10% NaCl concentration.

### Effect of lipopeptide on plant growth promotion

In this study, *Solanum lycopersicum* (tomato), *Pisum sativum* (pea), *Capsicum annuum* (chili pepper), and *Lactuca sativa* (lettuce) were examined to demonstrate the effects of biosurfactants, that showed significantly (P < 0.05) better effects on seed germination and plant growth promotion. Statistical data about seeds germination and plants growth obtained is shown in Additional file 1: Table S2. The best results for germination were obtained at higher concentrations (0.7 mg/mL) of lipopeptide tested. Among all seeds tested significant (P < 0.05) stimulation was observed for chili pepper seeds, showed almost double 51.7% germination at 0.5 g/100 mL in comparison to control 21.6% with MilliQ water. Similarly, tomato seeds showed 68.75% germination at 0.7 g/100 mL in comparison to control water (56.25%), while pea and lettuce seeds were affected to some extent as shown in (Additional file 1: Fig. S4a).

The applied biosurfactant treatments also augmented the dry biomass of plants. The plants that arose after treating with different concentrations of lipopeptide displayed higher biomass in comparison to control. Our current findings showed that a significant (P < 0.05) increase in weight was observed for chili pepper and lettuce at 0.7 g/100 mL of lipopeptide. Biomass exhibited by chilli and lettuce was 0.21 g and 0.25 g respectively, that is four times increase in relative to control 0.06 g of the seedling. Although for pea and tomato similarly a positive effect was noted with the addition of 0.7 g/100 mL of lipopeptide that significantly increase (P < 0.05) dry biomass almost double in relative to control (Additional file 1: Fig. S4b). Interestingly, in the present study positive effect of lipopeptide on dry biomass was observed for all seeds tested but maximum for chili pepper and lettuce.

Almost all surfactin concentrations tested showed an immense effect on root elongation. The plants treated with a higher concentration of lipopeptide 0.7 g/100 mL enhanced the root growth at maximum. Biosurfactant treatment signifies (P < 0.05) better elongation of seedling roots in lettuce, pea, and chili pepper almost two times greater than control. The tomato seedlings treated with lipopeptide also showed an increase in root development (Additional file 1: Fig. S4c).

**Fig. 5:**
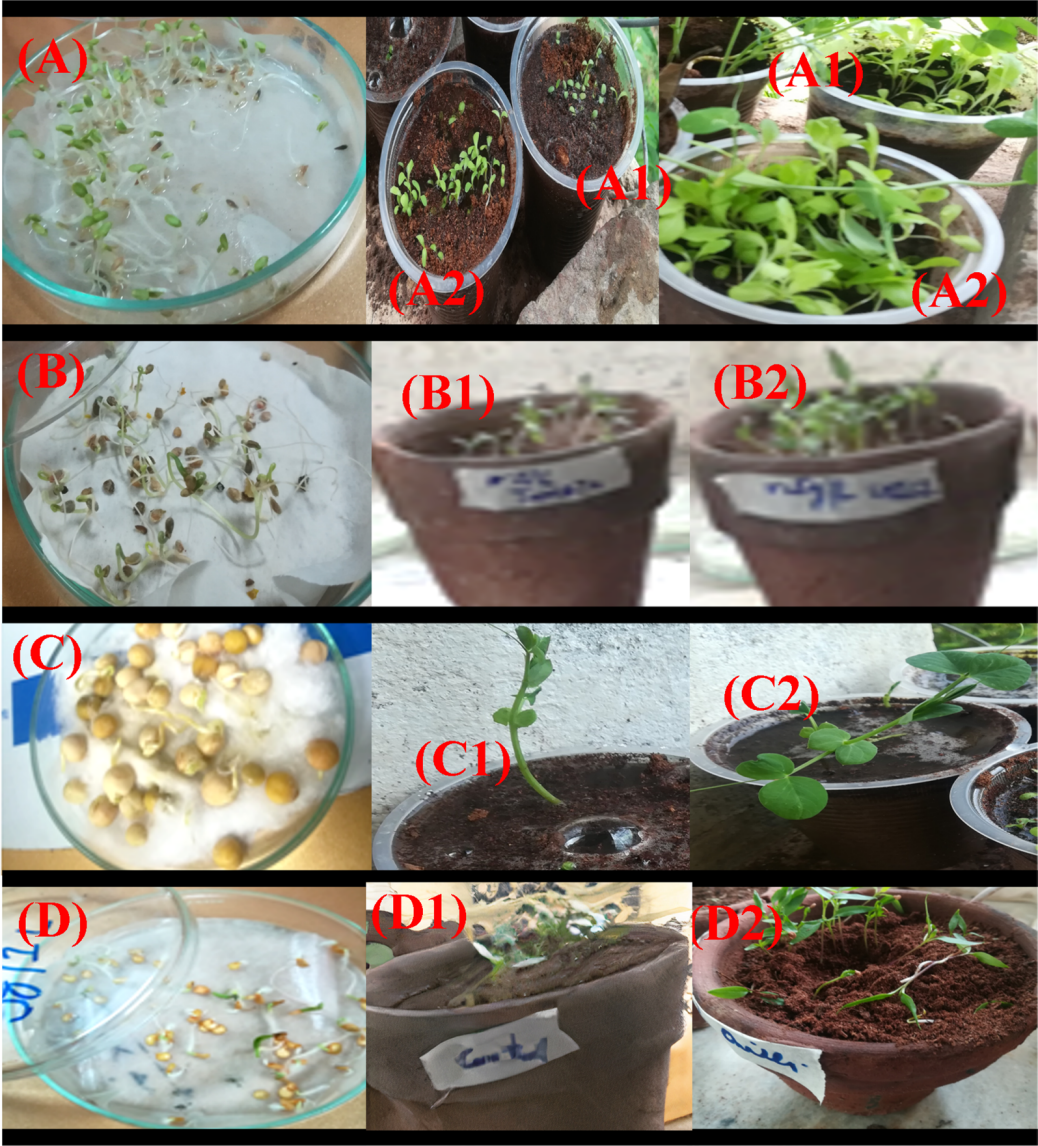
Effect of crude lipopeptide extract on seed germination of **A** lettuce, **B** tomato, **C** beans, and **D** chili The portion (**A**–**D1**) show untreated control plants and (**A**–**D2**) for plants treated with lipopeptide extract at 0.7 g/mL for 40 days

All lipopeptide concentrations tested showed significant effect on plant growth parameters. The chili pepper plants showed a significant (P < 0.05) difference in height 8.06 mm after treatment with 0.7 g/100 mL of lipopeptides almost double as compared to control (Additional file 1: Fig. S4d). Whereas lettuce plants showed a gradual increase in height with an increase in lipopeptide concentration. The effects of lipopeptide on seed germination and plant growth promotion are shown in Fig. 5.

### Bioremediation assay using shake flask fermentation

**Fig. 6:**
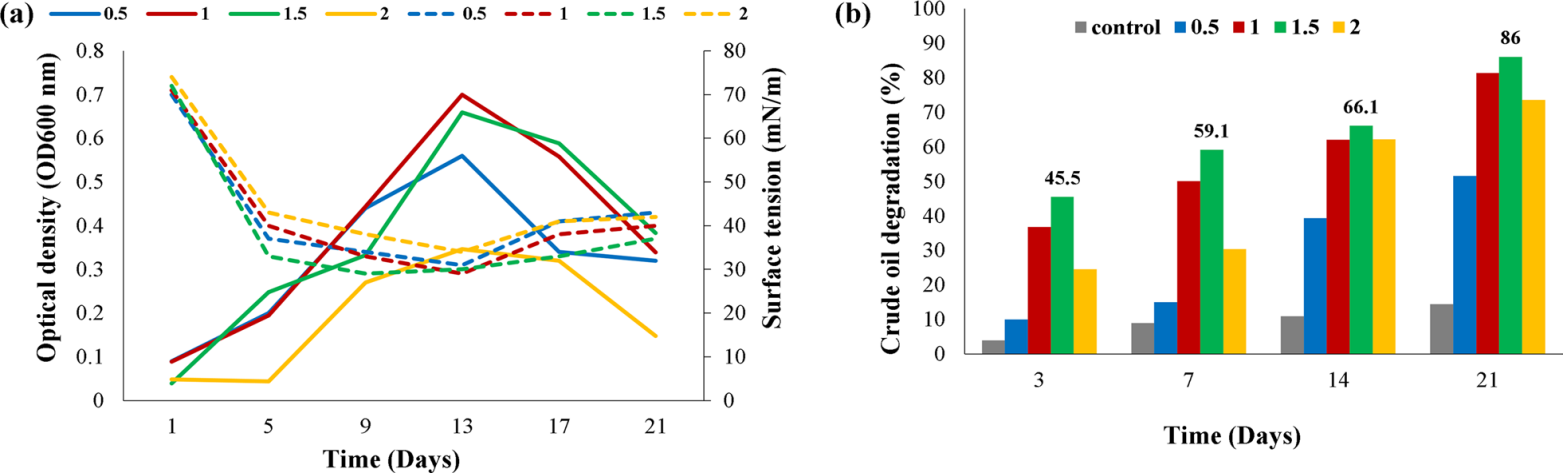
The growth of **a** *Bacillus subtilis* SNW3 on crude oil and MSM with surface tension reduction values for 21 days, plane lines for OD600 and dotted lines for SFT, and **b** quantity of crude oil degraded (%) by *Bacillus subtilis* SNW3 while growing on crude oil and MSM for 21 days

Biosurfactants are used to emulsify hydrocarbons with the reduction in surface tension, enhancement of water solubility, and increasing oil displacement from soil particles (Andrade Silva et al. 2014; Geetha et al. 2018). In the present study, the pattern for *Bacillus subtilis* SNW3 growth on crude oil and MSM revealed that there was an increase in microbial growth up to 13 days of incubation, whereas after that decline in growth was recorded. Observable effects on growth were observed with 1 and 1.5% of crude oil used. With increase in microbial growth the SFT value of the culture medium reduced from 72 to 29 mN/m, indicates the lipopeptide production by *Bacillus subtilis* SNW3 (Fig. 6a). The simultaneous microbial growth and crude oil biodegradation in culture broth media demonstrate utilization of various components of crude oil by *Bacillus subtilis* SNW3 (Patowary et al. 2017). However, we observed that maximum degradation 86% was achieved with 1.5% crude oil as compared to control (Fig. 6b; Additional file 1: Fig. S5c, d). Schematic diagram showing bacterial strain activity in degradation of crude oil recalcitrant hydrocarbons with simultaneously lipopeptide production (Additional file 1: Fig. S6).

### Bioremediation of crude oil in the soil through various design treatments

The current study revealed that lipopeptide produced by *Bacillus subtilis* SNW3 effectively removes crude oil from the soil. After applying different strategies, the residual crude oil content of each treatment showed different extents of biodegradation. It was revealed that the combined strategy used in T3 gave measurable remediation of crude oil as compared to other treatments tested. There was a gradual increase in bioremediation capacity with time, maximum after 90 days. T3 treated with *Bacillus subtilis* SNW3 cultured microorganisms and CFS containing lipopeptides (80.2%), show a significant difference from T0 control (11.6%) with distilled water. The better bioremediation results (73.2%) were obtained in T1 with the addition of lipopeptide than those obtained by T2 (63.8%) with the addition of biosurfactant producing *Bacillus subtilis* SNW3.

**Fig. 7:**
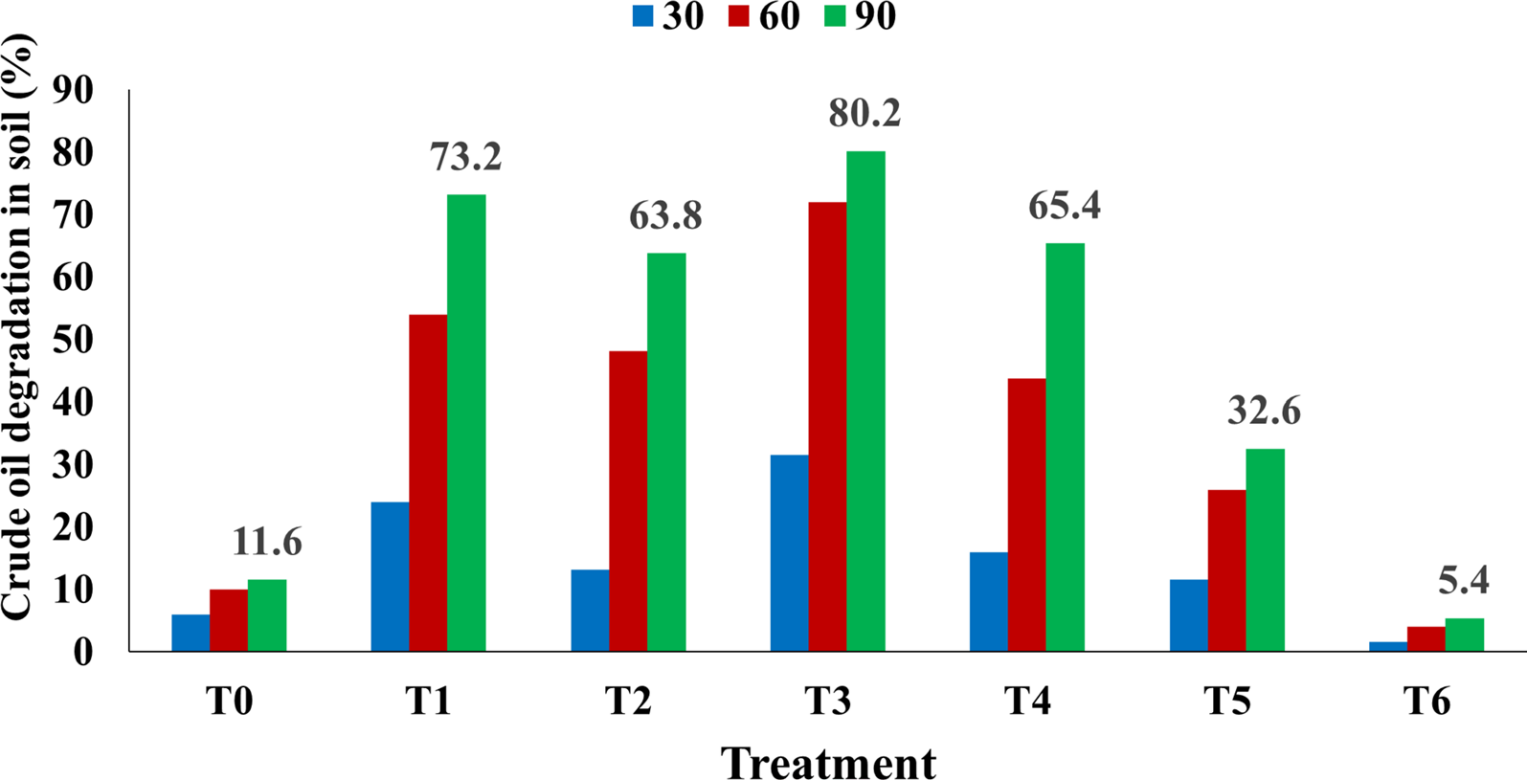
The percent degradation of crude oil contaminated soil through various design treatments from T1–T6 for 90 days

In the current study while making a comparison for bioremediation with chemical compounds it was observed that in T4, the addition of Tween 80 showed 65.4% degradation lower than those treated with biosurfactants. The oil reduction results obtained for T5 with fertilizer showed (32.6%) while the lowest degradation (5.4%) was observed for T6 using autoclaved soil (Fig. 7).

## Discussion

Biosurfactant production by using cost-effective substrates produced by *Bacillus subtilis* was previously studied by many researchers (Secato et al. 2016). An easy way to achieve cost-effective bioprocesses for biosurfactant production is by using a low-cost substrate. The use of waste frying oil as a sole source of carbon and energy for lipopeptide production by two *Bacillus* strains were previously reported by Md Badrul Hisham et al. (2019) that gave surface tension reduction values up to 36 mN/m. Our results showed that *Bacillus subtilis* SNW3 growing on 2% waste frying oil gave 38 mN/m reduction value are in line with previous findings. De Lima et al. (2009) reported rhamnose production by *Pseudomonas aeruginosa* PACL strain cultivating on waste frying soybean oil with 100% emulsification index, surface tension reduction up to 26.0 mN/m, and concentration of 3.3 g/L while in the current study 56.3% emulsification was observed with 2% waste frying oil. Research conducted by Abdel-Mawgoud et al. (2008) investigated surfactin production in a cost-effective manner with the use of 16% molasses and other trace elements that produce a surfactin yield of 1.12 g/L. However, it is also stated in many studies that the presence of hydrophobic substrate is essential for the production of biosurfactants (Santos et al. 2016). According to literature different types of oils e.g., vegetable oils, waste cooking oil, glycerol, glucose, and diesel were screened out for biosurfactant production by fungal species *M. circinelloides* that showed 11.7 cm ODA with the use of waste cooking oil as carbon source. In another study conducted by Hasanizadeh et al. (2017) for biosurfactant production showed maximum biosurfactant production with the use of 8% (v/v) waste cooking oil as a carbon source. Nitrogen is considered an important component of the medium used for biosurfactant production (Wu et al. 2008). In the literature, for higher biosurfactant yields number of nitrogenous compounds are listed that include yeast extract (Rodrigues et al. 2006a), beef extract, urea (Elazzazy et al. 2015), peptone, and meat extract (Gudiña et al. 2011). Urea was considered as a cheaper nitrogen source for significant lipopeptide production in comparison to sodium nitrate (Elazzazy et al. 2015; Farace et al. 2015; Ghribi and Ellouze-Chaabouni 2011). Yeast extract has been extensively selected in several studies (Marcelino et al. 2019). The yeast extract was used as a control medium as previously yeast extract was found as the most preferable substrate for significant biosurfactant production (Qazi et al. 2013). For instance, *L. paracasei* A20 preferred yeast extract as the significant medium for biosurfactant production followed by meat extract, while peptone was chartagorised as least important component of the medium (Gudiña et al. 2011).

It was reported previously that limitation in nitrogen concentration results in higher biosurfactant yield (Wu et al. 2019). The high carbon to nitrogen ratio (C/N) of the production medium (i.e., low level of nitrogen) limits bacterial growth, promoting cell metabolism for metabolites production (Nurfarahin et al. 2018). *P. aeruginosa* LBM10 in a culture medium containing soy bean oil as carbon source and NaNO_3_ as the source of nitrogen produce significant biosurfactant yield. Media composition with low nitrogen level produce (1.42 g/L) as compared to higher nitrogen concentrations with biosurfactant yield of 0.94 g/L (Prieto et al. 2008). The Kjeldahl or Dumas methods used for the evaluation of the crude protein in foods determine the total organic nitrogen of foods (Chandra-Hioe et al. 2018). The nitrogen content determination is crucial for the analysis of crude protein content. However, these provide rough assumptions as to the relative nitrogen and amino acid content differ between food proteins (Mæhre et al. 2018).

In the current study substrates were used in combination to increase biosurfactant production by reduce the price of culture media (Rufino et al. 2008). Slivinski et al. (2012) reported the use of okara obtained after processing ground soybeans as a substrate for surfactin by *Bacillus pumilus* UFPEDA 448. Zhu et al. (2013) also investigated the use of soybean flour as a substrate for surfactin production by *Bacillus amyloliquefaciens* XZ-173. To the best of our knowledge, for economical biosurfactant production, only a few studies are conducted by using soybean, but no single study is present that investigated the use of white beans powder as a substrate for low-cost production. The concentration of crude lipopeptide produced was (about 1.17 g/L), closed to other reported values for biosurfactant production using cost-effective substrates. The cost required for the preparation of one liter of optimized low-cost media in the current study is 0.078 EUR, which is just 0.8% of one-liter synthetic yeast extract media cost 10.5 EUR. Hence utilizing these cost-effective nonconventional media instead of synthetic yeast extract contribute to a 99% reduction in cost required for medium preparation.

It was reported by Samak et al. (2020) that 30°C is the optimum temperature for biosurfactant production that is in  correspondence with results obtained in the current study. Our findings showed that no significant lipopeptide was produced at a static condition that might be due to lack of oxygenation (Santos et al. 2014). In a previous study conducted by Hemlata et al. (2015) for biosurfactant production by *Stenotrophomonas maltophilia* NBS-11 showed maximum production at pH 7. Urea and ammonium nitrate have been already used and reported in the literature as a very cost-effective nitrogen source for biosurfactant production by *Candida* spp. (Alwaely et al. 2019).The study conducted by Medeot et al. (2017) showed a high yield of biosurfactants (1.7 mg/mL) while using NH_4_NO_3_ and glucose as substrate for production by *Bacillus amyloliquefaciens* MEP218. In the same way, the combination of sucrose and NH_4_NO_3_ was reported by Fernandes et al. (2016), gave high yield of biosurfactant (0. 2 g/L) by *Bacillus subtilis* RI4914. Moreover, a study conducted for biosurfactant production showed optimum yield with 0.3% sodium nitrate by *Pseudoxanthomonas* sp. G3 (Purwasena et al. 2020). Current findings also showed maximum lipopeptide yield while using carbon nitrogen substrates in combination.

Surfactin acts as quorum-sensing molecule that provides a potential tool for the regulation of fermentation (Gupta et al. 2017), while carbon metabolism regulates the balance between the products and biomass. Biosurfactant production occurs frequently during a stationary stage of the cell growth after depletion of the nitrogen source (Onwosi and Odibo 2012). As a result of the current study, the final optimized carbon–nitrogen combination of the media significantly produced maximum biosurfactant with biomass and crude lipopeptide yield of 4.6 and 1.17 g/L respectively. Recently, Phulpoto et al. (2020) reported glycerol and NH_4_NO_3_ as combined C/N media for surfactin production that significantly produce biomass and crude biosurfactant yield of 4140 and 1255 mg/L, respectively. Similarly, it was reported by Lu et al. (2016), that for fengycin production through *Bacillus amyloliquefaciens* fmb-60 biomass yield could be a significant factor. In *Bacillus amyloliquefaciens* BZ-6 (Liu et al. 2012), *Bacillus amyloliquefaciens* MEP218 (Medeot et al. 2017), *Bacillus amyloliquefaciens* fmb-60 (Lu et al. 2016) and *Bacillus subtilis* strains (Makkar et al. 2011), a direct correlation was reported between lipopeptide production and biomass yield. Primary characterization for biosurfactant produced by *Bacillus subtilis* SNW3 was carried out by using TLC using surfactin from sigma as standard. Here, our results for TLC of crude biosurfactant sample indicated lipopeptide nature of biosurfactant product through the presence of surfactin with an Rf value of 0.68. These findings are consistent with other reported studies, where Rf value of 0.76 was observed by Ramyabharathi et al. (2018) for surfactin produced by *Bacillus subtilis.* Results obtained from previous findings also showed Rf values of 0.09, 0.3, and 0.75 for fengycin, iturin, and surfactin respectively using *Bacillus subtilis* UMAF6619, UMAF6614, UMAF8561, UMAF6639, and *Bacillus amyloliquefaciens* PPCB004 (Arrebola et al. 2010). FTIR results obtained were following TLC. The chemical structure of biosurfactant produced by *Bacillus subtilis* SNW3 was revealed by analyzing the crude extract using fourier transform infrared spectroscopy. FTIR analysis of crude biosurfactant produced by *Bacillus subtilis* SNW3 showed that it contains alcohols and carboxylic acids (lipids) and peptide moieties (proteins) that indicate lipopeptide nature of biosurfactant. A similar pattern of FTIR aliphatic and peptide moieties was reported for the presence of lipopeptides by Kiran et al. (2017). The observed pattern of IR spectrum was very similar to the spectrum obtained by de Faria et al. (2011) who reported the appearance of the stretch at 1721 cm^−1^ that indicates the presence of lactone carbonyl group. Similar FTIR absorption spectra were reported in the literature for lipopeptide (Pereira et al. 2013).

Lipopeptide produced during the current study not only provides potential antibacterial activity but also renders bacteria more susceptible to the available antibiotics. Biosurfactants could be a suitable substitute for antimicrobial compounds and synthetic medicines and might be used as efficient therapeutic agents (Gudiña et al. 2013). The antimicrobial effect of biosurfactants is due to their potential to form pores inside cell membranes (Gudiña et al. 2010) this characteristic might increase the effectiveness of antibiotics. Sambanthamoorthy et al. (2014) revealed antimicrobial activities against *A. baumannii*, *E. coli*, and *S. aureus* at a concentration of 25–50 mg/mL. In our findings lipopeptide showed antimicrobial effect at a lower concentration of 10 mg/mL that showed more effectiveness of the lipopeptide product. These results are consistent with previous studies which suggest a synergistic effect of biosurfactant with antibiotics (Joshi-Navare and Prabhune 2013; Rivardo et al. 2011). The promising feature of natural antimicrobial peptides is their low toxicity and slow microbial resistance emergence rate as compared to the current antibiotics (Wang et al. 2019). Our findings suggested that lipopeptides could extend the clinical use of current antibiotics. Domhan et al. (2018) reported lipopeptides as novel antimicrobial agents against resistant microbial pathogens with favorable pharmacokinetics and enhanced antibacterial activity.

The critical micelle concentration (CMC) is the minimum biosurfactant concentration needed to achieve the lowest surface tension value at which micellar aggregates formation starts (Ma et al. 2016). CMC is an important characteristic of surface-active agents for evaluation of their interfacial activity (Zhou et al. 2019b). The CMC of crude lipopeptide produced by *Bacillus subtilis* SNW3 was found to be ≤ 0.58 mg/mL, significant as compared to 2.7 mg/mL (Ghasemi et al. 2019). These results were also efficient as compared to commonly used synthetic surfactants sodium dodecyl sulfate (SDS) that attains CMC value at 2100 mg/L (Chen et al. 2006).

After biosurfactant production purification strategies account for near 60% of the total cost (Banat et al. 2010). Taking into consideration the industrial economic value, most the biosurfactants are required either in crude form or in form of broth preparations (Banat et al. 2010). Lipopeptide produced by *Bacillus subtilis* SNW3 exhibits excellent stability over an extensive range of pH (1–11), salinity (1–8%), temperature (20–121°C), and even after autoclaving. The decrease in stability of biosurfactants at acidic conditions might be due to the protonation of negative polar ends of surfactin molecules (Gogoi et al. 2016). A previous study conducted by Purwasena et al. (2019) showed biosurfactant stability with emulsification at a high temperature of 120 °C, pH of 4–10, and NaCl concentration of 10% (w/v) that are in accordance with the current study. Moussa et al. (2013) showed biosurfactant stability at 20–120°C produced by *Bacillus methylothrophicus* and *Rhodococcus equi* strains. Different studies showed reduced biosurfactant stability under alkaline conditions. Several other studies have been reported about the stability of the biosurfactants at high salinity and temperature (Rodrigues et al. 2006b; Gudina et al. 2010). Significant stability of lipopeptide was found at high salinity and temperature and our findings were inconsistent with previous studies (Das and Kumar 2018; Hentati et al. 2019; Purwasena et al. 2019). Surfactin with excellent stability at wide range of temperature, pH and salinity widens its applicability in several industrial sectors e.g. food, pharmaceuticals, detergents, agricultural and bioremediation (Fenibo et al. 2019).

In the modern agricultural field use of bacterial biosurfactants plays an important role as they are eco-friendly and affordable (Hafeez et al. 2019; Muthusamy et al. 2008). Lipopeptides derived from bacterial strains are eco-friendly, less toxic, with more stability in harsh environments and highly biodegradable as compared to their synthetic counterparts (Lima et al. 2011). The genera *Bacillus* and *Pseudomonas* proved as e major producers of biosurfactant molecules (Hussain and Khan 2020; Zhou et al. 2019a). Lipopeptide produced by *Bacillus subtilis* SNW3 had a noticeable effect on seed germination and plant growth promotion that becomes more prominent with the increase in concentration. Our findings showed significant increase in seed germination of all four species tested most prominent for chilli and tomato. This increase in germination might be due to the reason that biosurfactant increases the permeability of seed coat to water that indirectly makes quicker the metabolic processes inside seeds (Kaur et al. 2017). The applied lipopeptide treatments augmented the dry biomass of all seeds tested, maximum for chilli pepper and lettuce. Similar results with an increase in plant biomass were observed by Liu et al. (2014). This increase in plant biomass might be due to the enhanced production of phytohormones and improved mineral solubilization in soil (Das and Kumar 2016). Almost all biosurfactant concentrations tested showed an immense effect on root elongation with better elongation in lettuce, pea and chilli. Enhanced plants root elongation could be due to decrease strength of wrapping tissues and seed coating that favors root development (da Silva et al. 2015). Another reason for the increase in root development by applying biosurfactants could be due to minimizing anaerobiosis conditions in the soil (Shukry et al. 2013). In current study significant effect was observed on plant growth promotion more prominent for chilli plants as compared to control. It was demonstrated by Cawoy et al. (2014) that surfactin by *Bacillus* isolates provokes concentration dependent induce systemic resistance (ISR). Surfactin act as a signaling molecule that provokes cannibalism and the formation of a matrix (López et al. 2009b). The improved plant development with biosurfactants ciould be incresae nutrients bioavailability and emulsification of hydrophobic compounds (Marchut-Mikolajczyk et al. 2018). Several researchers have reported the biosurfactant effect on seed germination, but to our knowledge, no previous study is available about current vegetable plants. Biopreparations are widely used nowadays for the enhanced seed quality and improved plant germination in contaminated soil (Mukherjee et al. 2006). However, some research gaps are still required to be filled about mechanisms followed by biosurfactants concerning enhanced growth and development of plants.

Lipopeptides are mostly applied in the biomedical field and only a few reports are present that showed the success story of lipopeptides in bioremediation of oil-polluted environments. In recent years, the use of biosurfactants for the treatment of oil-contaminated soil is increased (Karlapudi et al. 2018). Indigenous microbes that are normally present in oil-contaminated soil are mainly involved in the biodegradation of oil pollutants (Iwai et al. 2011; Lee et al. 2018). Through introducing biosurfactant-producing bacteria in the contaminated environment results in enhanced bioremediation utilizing solubilization, mobilization, and emulsification of hydrocarbons (Nievas et al. 2008). Crude oil is a complex mixture of aliphatic and aromatic hydrocarbons that inhibits the uptake of carbon sources required for metabolism and growth (Das and Chandran 2011). Many reports are present about the efficacy of biosurfactants produced by *Bacillus* species in oil recovery methods, bioremediation processes and industrial sectors (Greenwell et al. 2016; Ismail et al. 2013; Pereira et al. 2013).

Data obtained from our findings suggested *Bacillus subtilis* SNW3 as a potential bioremediation agent as compared to previously reported biodegradation studies (Sathishkumar et al. 2008). Bioremediation experiments conducted by Kumari et al. (2012) showed degradation percent of 49.5 and 60.6% for total petroleum hydrocarbons (TPH) by *Rhodococcus* sp. NJ2 and *Pseudomonas* sp. BP10 respectively. In our study more significant biodegradation of 86% was observed after 21 days. Al-Wasify and Hamed (2014) explained that *P. aeruginosa* reveal about 77.8% of maximum degradation after an incubation period of 28 days. Studies reported bioremediation of 49–54% for crude oil-polluted environments (Bordoloi and Konwar 2008) and more than 85% for diesel oil-contaminated sand (Silva et al. 2010).

Biosurfactant produced by *S. marcescens* UEO15 confirmed 59% and 78% degradation of kerosene and crude oil, in comparison of 25% and 10% with distilled water used as control (Elemba et al. 2010). Our study showed 86% efficacy in comparison to previous findings suggesting it as a more suitable bioremediation component in environmental sectors.

Tween 80 is suitable for remediation of contaminated soil because of its low cost as compared to other non-ionic surfactants (Bautista et al. 2009), most successfully reported for polycyclic aromatic hydrocarbons PAHs (Gong et al. 2015). In the current study, T4 showed a 65.4% degradation rate that might be due to acidic conditions of soil that is unsuitable for microbial growth (Liu et al. 2010). Our findings suggested that treatments with biosurfactants enhanced the degradation rate employed it as a better bioremediation agent. Mishra and Singh (2012) reported that among degradative enzymes alkane hydroxylase produced by *Rhodococcus* sp. NJ2 and *P. aeruginosa* PSA5 result in degradation of n-hexadecane. Genes involved in the production of these degradative enzymes are reported in previous studies (de Gonzalo et al. 2016). Previous studies have reported the fertilizer as treatment to check the effects of NPK on the biodegradation of hydrocarbons (Pelletier et al. 2004). Nutrients especially phosphorus, nitrogen, and in some cases, iron are very essential ingredients for successful biodegradation of hydrocarbon pollutants (Adams et al. 2015).

Moreover, bioremediation technology is considered to be non-invasive and quite cost-effective (Azubuike et al. 2016; Kumar and Yadav 2018). Biodegradation through microorganisms signifies one of the principal mechanisms by which petroleum and other hydrocarbon pollutants can be removed from the environment (Al-Hawash et al. 2018; Das and Chandran 2011) and is cheaper than other remediation technologies (da Rocha Junior et al. 2019).

The current study demonstrated the use of cost-effective media for lipopeptide production by *Bacillus subtilis* SNW3. The possibility of utilizing waste frying oil in combination with white beans might be proved to be efficient to substitute yeast extract media and worthwhile for its industrial-scale production. The lipopeptides obtained exhibited potential emulsifying and surface tension reducing capabilities with strong stability at a wide range of pH, temperature, and salinity. In addition, lipopeptide produced showed higher potential for seed germination and plant growth promotion of *Capsicum annuum*, *Lactuca sativa*, *Solanum Lycopersicum*, *Pisum sativum*, and removal of crude oil from contaminated soil, suggesting its potential applications in environmental and agriculture sectors.

## Supplementary Information

**Additional file 1: Figure S1.** Effect of cultural conditions on lipopeptide production by *Bacillus subtilis* SNW3. (a) Temperature (b) inoculum (c) agitation and (d) pH, error bars represent ± standard deviation of triplicate values. **Figure S2.** Antibacterial activity of lipopeptide produced by *Bacillus subtilis* SNW3 against *Escherichia coli* and *Salmonella typhi* by using agar well diffusion assay. The error bars represent ± standard deviation of triplicate values. **Figure S3.** Lipopeptide characterization by (a) critical micelles concentration (CMC) and (b) critical micelles dilution (CMD); produced by *Bacillus subtilis* SNW3 in relation to SFT measurement under optimized conditions. **Figure S4.** Effect of crude lipopeptide extract obtained from *Bacillus subtilis* SNW3 on (a) germination of seeds (b) dry biomass of plant (c) root length and (d) height of plants. The error bars represent ± standard error of triplicate values. **Figure S5.** Results obtained for lipopeptide produced on optimized media (a) oil displacement activity in crude oil, (b) Emulsification activity (E24) up to 70%) (c) screening of *Bacillus subtilis* SNW3 for growth and bioremediation with 1.5% crude oil in uninoculated control and sample after 21 days (d) extraction of crude oil media with hexane after 21 days from uninoculated control and sample through gravimetric analysis. **Figure S6.** Schematic diagram showing bacterial strain activity in degradation of crude oil recalcitrant hydrocarbons with simultaneously lipopeptide production. **Table S1.** Analysis of total organic carbon (TOC) and total organic nitrogen (TON) content of the substrate tested. **Table S2.** Statistical Mean (M), Std. Deviation (SD), Std. Error (SE) and P value for relative seed germination, dry biomass, root length and plant height at four different concentrations of lipopeptide produced by *Bacillus subtilis* SNW3 used for four different plant species.

## Data Availability

The data used to support the findings of this study are available from the corresponding author upon request.

## Abbreviations

ATCC: American type culture collection
BCCM: Belgian coordinated collections of microorganisms
CMC: Critical micelle concentration
CMD: Critical micelle dilution
CFS: Cell-free supernatant
E24: Emulsification ability
FTIR: Fourier-transform infrared spectroscopy
HCl: Hydrochloric acid
HCN: Hydrogen cyanide
IAA: Indole acetic acid
ISR: Induced systemic resistance
Mol.: Molasses
MSM: Mineral salt medium
NaCl: Sodium chloride
NARC: National Agricultural Research Council
OD: Optical density
ODA: Oil displacement activity
P.P.P: Potato peels powder
PAHs: Polycyclic aromatic hydrocarbons
PGPR: Plant growth-promoting rhizobacteria
Rf: Retention factor
SDS: Sodium dodecyl sulfate
SFT: Surface tension
TLC: Thin-layer chromatography
TOC: Total organic carbon
TON: Total organic nitrogen
TPH: Total petroleum hydrocarbons
W.B.P: White beans powder
W.F.O: Waste frying oil
Y.E: Yeast extract
