# Supplementary material for: Low-cost production and application of lipopeptide for bioremediation and plant growth by Bacillus subtilis SNW3
Source: AMB Express. 2021 Dec 11;11:165. doi: 10.1186/s13568-021-01327-0 (PMC8665955; doi:10.1186/s13568-021-01327-0)
Supplement: Supplementary file 1 — Additional file 1: Figure S1. Effect of cultural conditions on lipopeptide production by Bacillus subtilis SNW3. (a) Temperature (b) inoculum (c) agitation and (d) pH, error bars represent ± standard deviation of triplicate values. Figure S2. Antibacterial activity of lipopeptide produced by Bacillus subtilis SNW3 against Escherichia coli and Salmonella typhi by using agar well diffusion assay. The error bars represent ± standard deviation of triplicate values. Figure S3. Lipopeptide characterization by (a) critical micelles concentration (CMC) and (b) critical micelles dilution (CMD); produced by Bacillus subtilis SNW3 in relation to SFT measurement under optimized conditions. Figure S4. Effect of crude lipopeptide extract obtained from Bacillus subtilis SNW3 on (a) germination of seeds (b) dry biomass of plant (c) root length and (d) height of plants. The error bars represent ± standard error of triplicate values. Figure S5. Results obtained for lipopeptide produced on optimized media (a) oil displacement activity in crude oil, (b) Emulsification activity (E24) up to 70%) (c) screening of Bacillus subtilis SNW3 for growth and bioremediation with 1.5% crude oil in uninoculated control and sample after 21 days (d) extraction of crude oil media with hexane after 21 days from uninoculated control and sample through gravimetric analysis. Figure S6. Schematic diagram showing bacterial strain activity in degradation of crude oil recalcitrant hydrocarbons with simultaneously lipopeptide production. Table S1. Analysis of total organic carbon (TOC) and total organic nitrogen (TON) content of the substrate tested. Table S2. Statistical Mean (M), Std. Deviation (SD), Std. Error (SE) and P value for relative seed germination, dry biomass, root length and plant height at four different concentrations of lipopeptide produced by Bacillus subtilis SNW3 used for four different plant species. [file 13568_2021_1327_MOESM1_ESM.docx]

**Supplementary Figure captions:**

**Figure S1.** Effect of cultural conditions on lipopeptide production by *Bacillus subtilis* SNW3 (a) Temperature (b) inoculum (c) agitation and (d) pH, error bars represent ± standard deviation of triplicate values.

**Figure S2.** Antibacterial activity of lipopeptide produced by *Bacillus subtilis* SNW3 against *Escherichia coli* and *Salmonella typhi* by using agar well diffusion assay. The error bars represent ± standard deviation of triplicate values.

**Figure S3.** Lipopeptide characterization by (a) critical micelles concentration (CMC) and (b) critical micelles dilution (CMD); produced by *Bacillus subtilis* SNW3 in relation to SFT measurement under optimized conditions.

**Figure S4.** Effect of crude lipopeptide extract obtained from *Bacillus subtilis* SNW3 on (a) germination of seeds (b) dry biomass of plant (c) root length and (d) height of plants. The error bars represent ± standard error of triplicate values.

**Figure S5.** Results obtained for lipopeptide produced on optimized media (a) oil displacement activity in crude oil, (b) Emulsification activity (E24) up to 70%) (c) screening of *Bacillus subtilis* SNW3 for growth and bioremediation with 1.5% crude oil in uninoculated control and sample after 21 days (d) extraction of crude oil media with hexane after 21 days from uninoculated control and sample through gravimetric analysis.

**Figure S6.** Schematic diagram showing bacterial strain activity in degradation of crude oil recalcitrant hydrocarbons with simultaneously lipopeptide production.

**Figure S1.**


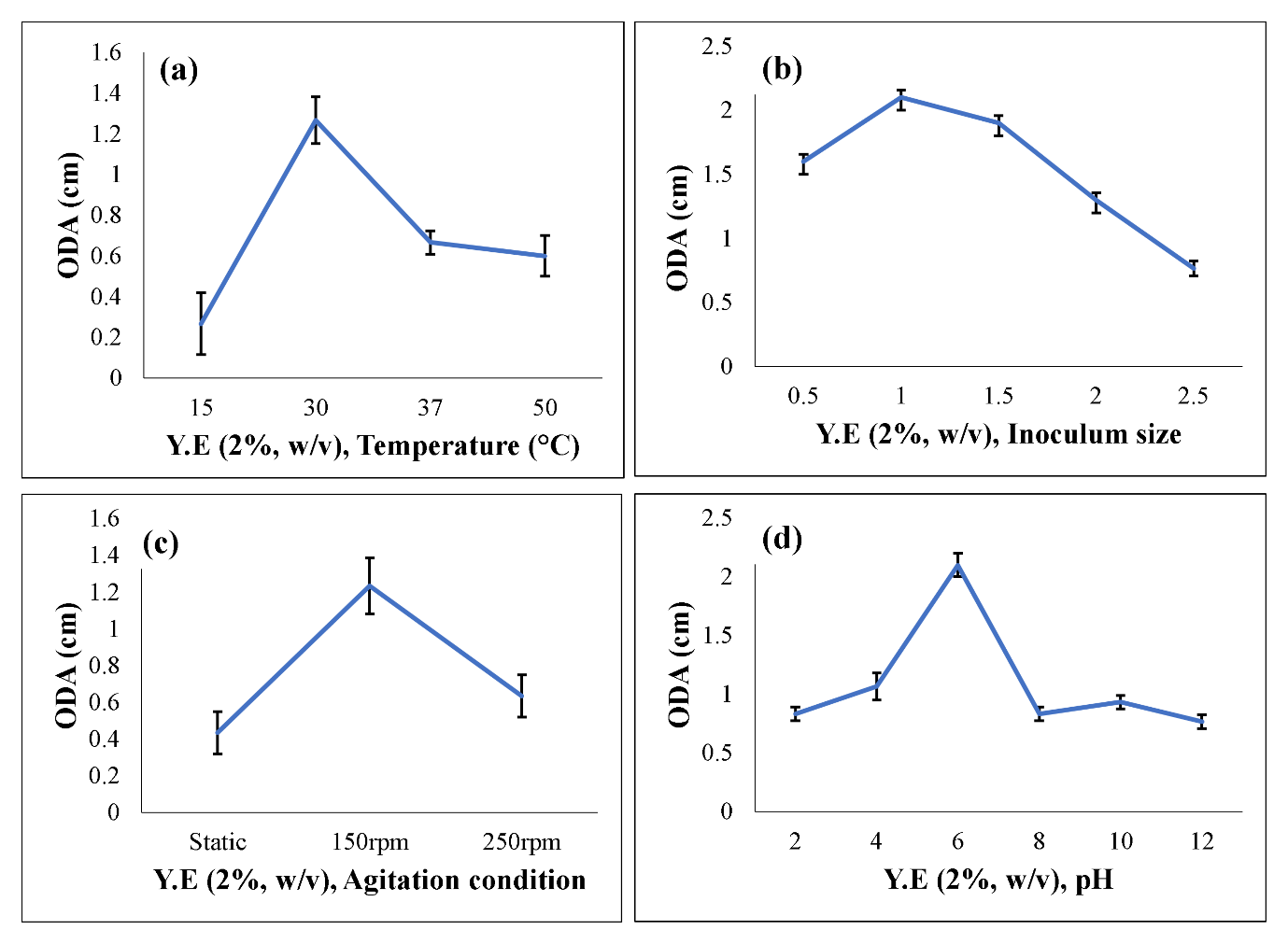


**Figure S2.**


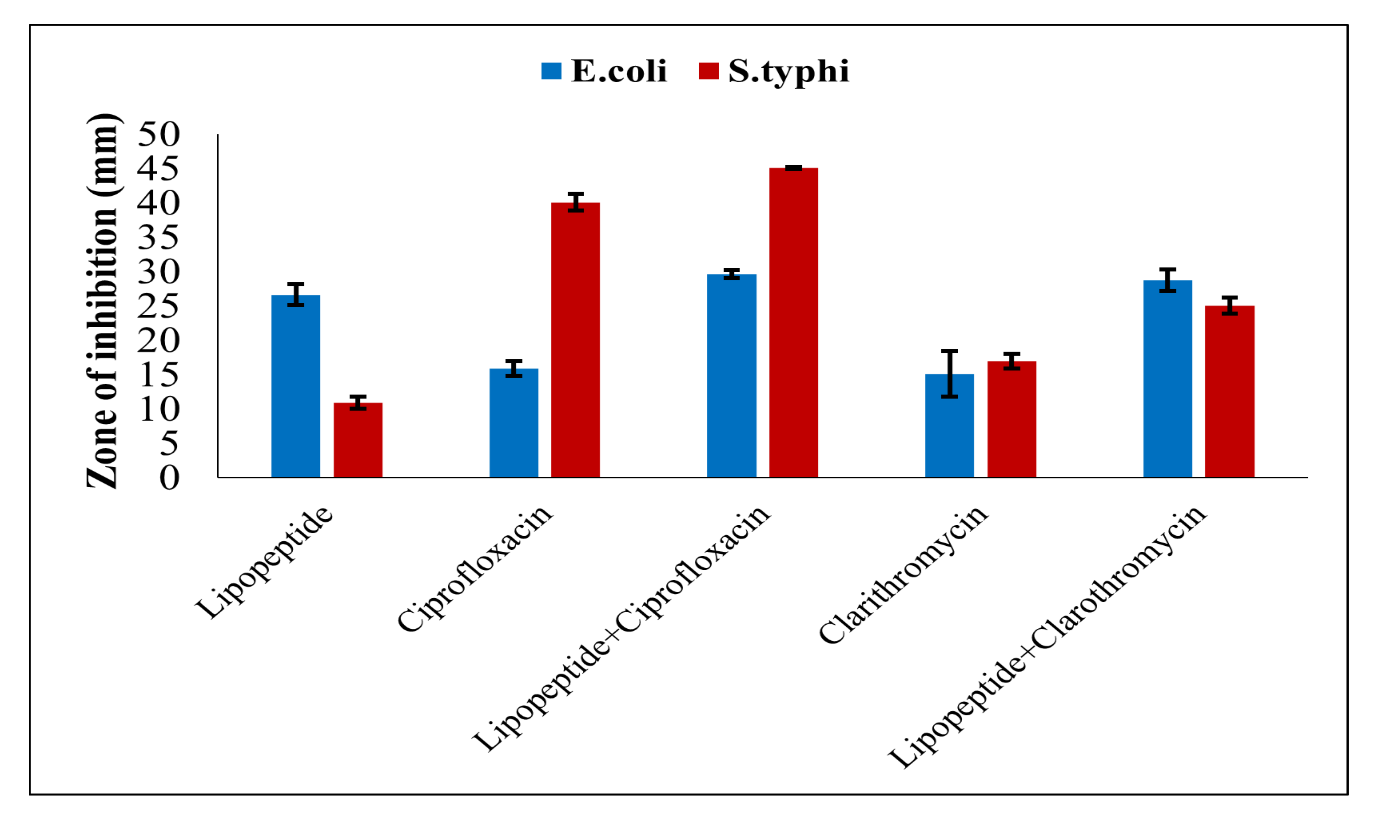


**Figure S3.**


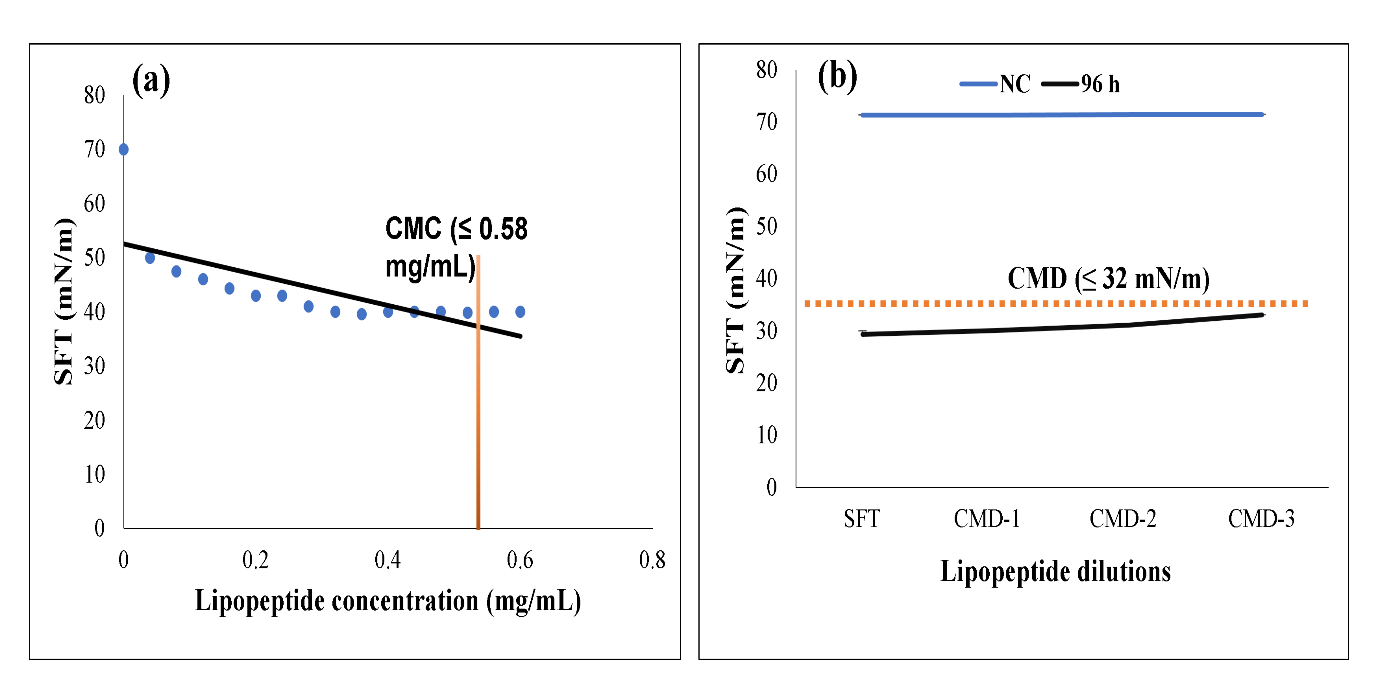


**Figure S4.**


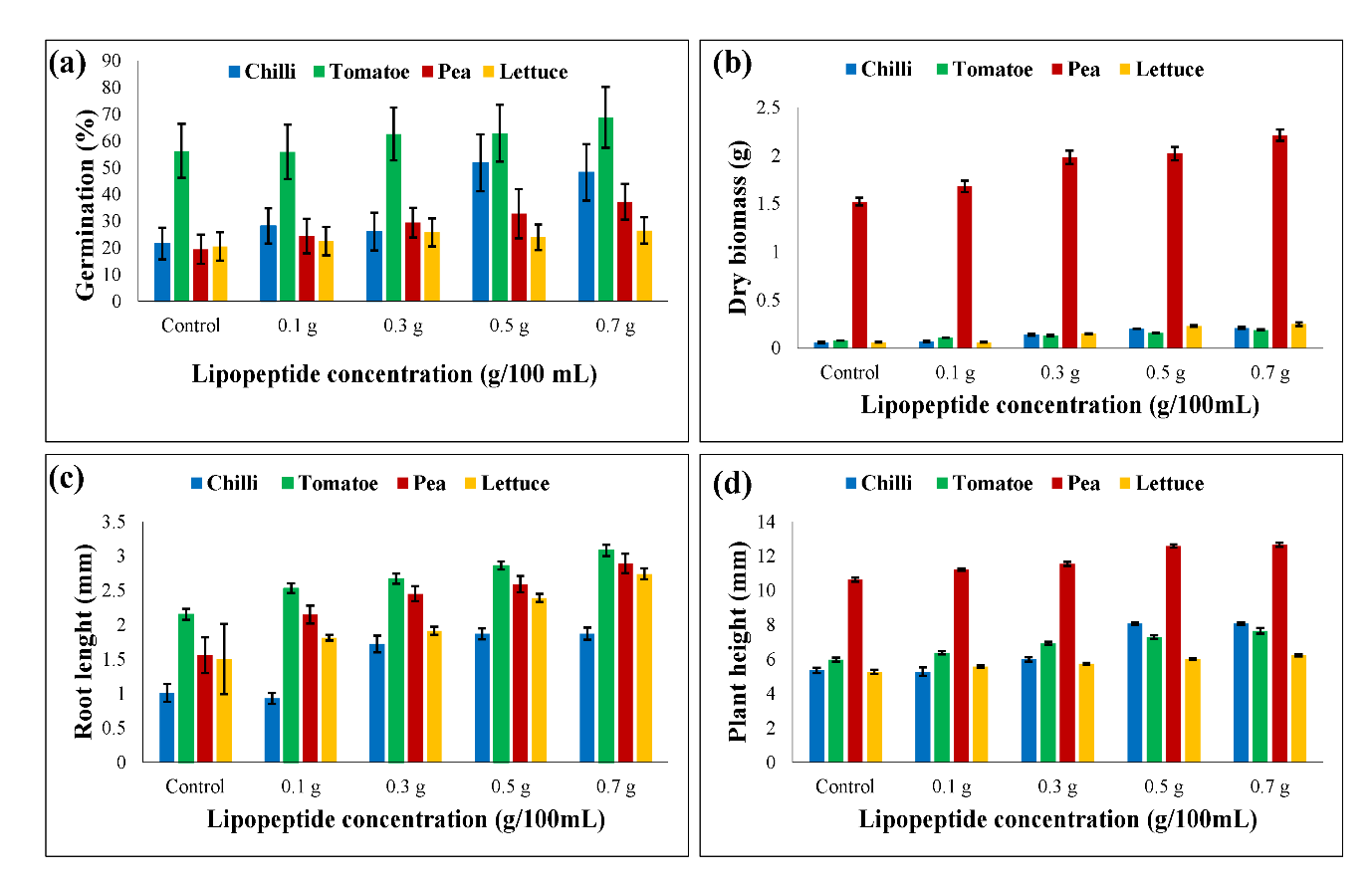


**Figure S5.**


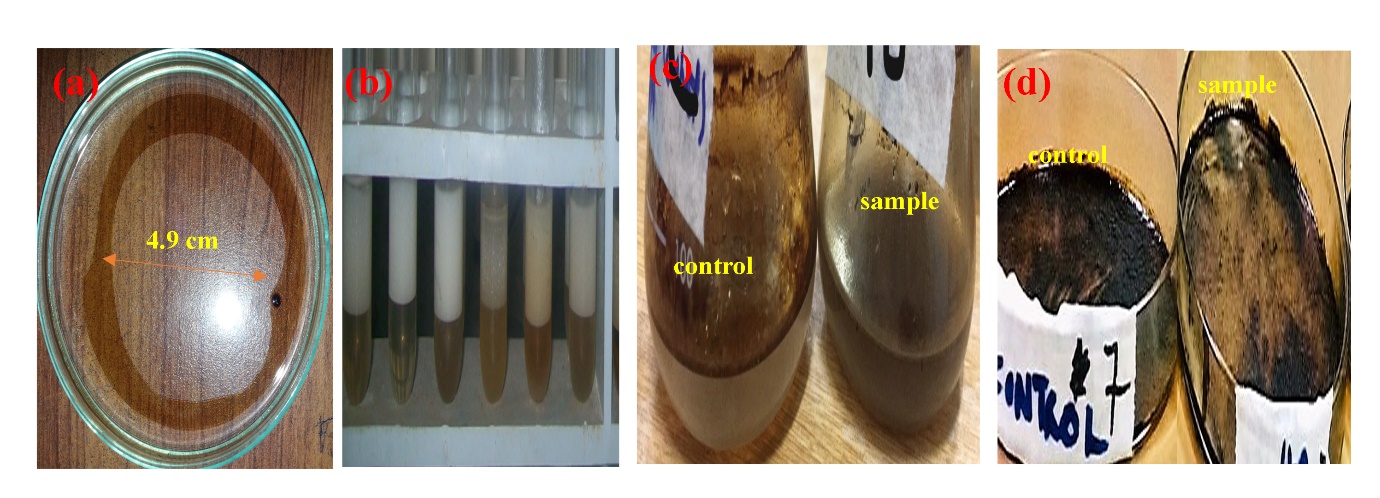


**Figure S6.**


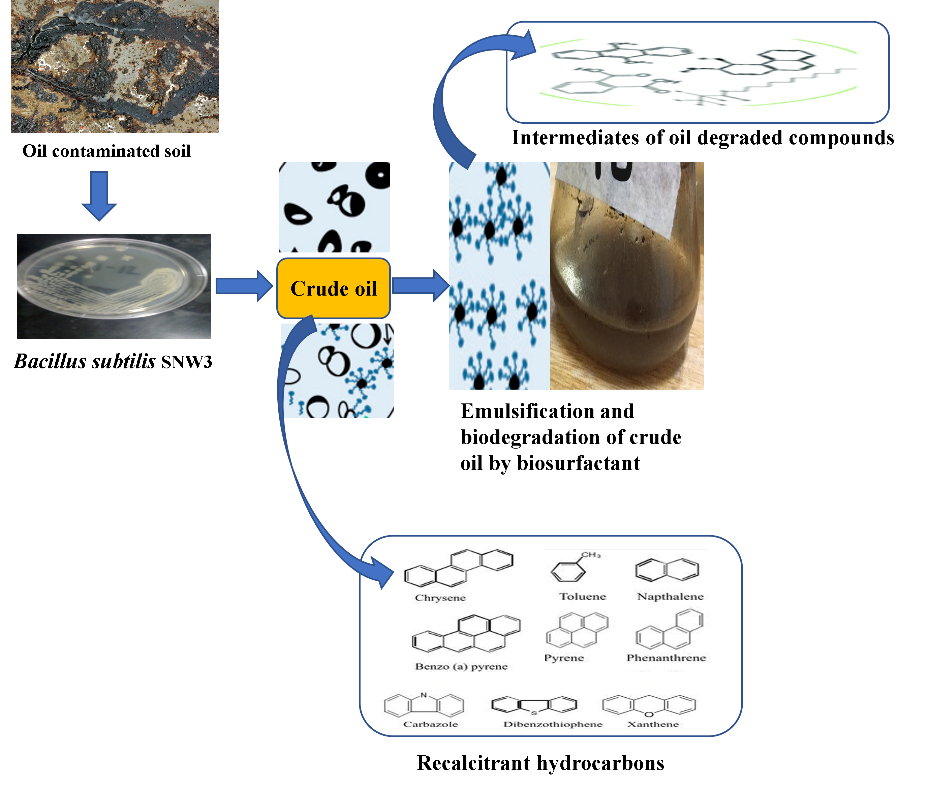


**Table S1.** Analysis of total organic carbon (TOC) and total organic nitrogen (TON) content of the substrate tested.

**Table S2.** Statistical Mean (M), Std. Deviation (SD), Std. Error (SE) and P value for relative seed germination, dry biomass, root length and plant height at four different concentrations of lipopeptide produced by *Bacillus subtilis* SNW3 used for four different plant species.

**Table S1.**

|  | Solid sample |  | Liquid sample |  |  |
| --- | --- | --- | --- | --- | --- |
|  | Dumas method | Dumas  method | TOC analyzer  Combustion method | Kjeldahl method | |
| Substrates | Total organic carbon (mg/ g) | Total organic nitrogen (mg/ g) | Total organic carbon (mg/L) | Kjeldahl nitrogen  (mg/L) | |
| Potato peels powder | 182 | 19 | 370 | 38.8 | |
| White beans powder | 139 | 20 | 1400 | 198 | |
| Yeast extract | 314 | 97 | 3200 | 988 | |

**Table S2.**

|  |  | **Control** | | | | **0.1g** | | | | **0.3g** | | | | **0.5g** | | | | **0.7g** | | | |
| --- | --- | --- | --- | --- | --- | --- | --- | --- | --- | --- | --- | --- | --- | --- | --- | --- | --- | --- | --- | --- | --- |
|  |  | **M** | **SD** | **SE** | **P** | **M** | **SD** | **SE** | **P** | **M** | **SD** | **SE** | **P** | **M** | **SD** | **SE** | **P** | **M** | **SD** | **SE** | **p** |
| **Root length** | **Chilli** | 1.01 | .43 | .13 | .00 | .93 | .32 | .08 | .00 | 1.72 | .49 | .12 | .00 | 1.87 | .45 | .08 | .00 | 1.87 | .53 | .09 | .00 |
|  | **Tomato** | 2.15 | .46 | .08 |  | 2.53 | .38 | .07 |  | 2.67 | .42 | .074 |  | 2.86 | .36 | .06 |  | 3.08 | .51 | .08 |  |
|  | **Pea** | 1.56 | 1.05 | .26 |  | 2.15 | .42 | .13 |  | 2.45 | .36 | .11 |  | 2.59 | .49 | .12 |  | 2.89 | .53 | .14 |  |
|  | **Lettuce** | 1.50 | .29 | .51 |  | 1.81 | .23 | .04 |  | 1.91 | .34 | .06 |  | 2.39 | .34 | .06 |  | 2.74 | .46 | .08 |  |
| **% Germination** | **Chilli** | 21.6 | 15.47 | 5.85 | .00 | 28.21 | 17.31 | 6.54 | .02 | 26.07 | 18.65 | 7.05 | .00 | 51.79 | 28.16 | 10.64 | .03 | 48.21 | 27.72 | 10.48 | .02 |
|  | **Tomato** | 56.25 | 24.69 | 10.08 |  | 55.83 | 24.88 | 10.16 |  | 62.50 | 24.19 | 9.87 |  | 62.92 | 26.00 | 10.61 |  | 68.75 | 27.92 | 11.39 |  |
|  | **Pea** | 19.43 | 13.25 | 5.49 |  | 24.40 | 15.84 | 6.47 |  | 29.42 | 13.42 | 5.48 |  | 32.75 | 22.34 | 9.12 |  | 37.20 | 16.39 | 6.69 |  |
|  | **Lettuce** | 20.50 | 12.93 | 5.28 |  | 22.50 | 13.11 | 5.35 |  | 25.83 | 12.82 | 5.23 |  | 24.00 | 11.69 | 4.77 |  | 26.50 | 12.18 | 4.97 |  |
| **Plant height** | **Chilli** | 5.35 | .61 | .16 | .00 | 5.27 | .94 | .25 | .00 | 5.99 | .54 | .13 | .00 | 8.08 | .39 | .07 | .00 | 8.06 | .39 | .07 | .00 |
|  | **Tomato** | 5.97 | .66 | .12 |  | 6.37 | .50 | .09 |  | 6.92 | .43 | .08 |  | 7.28 | .61 | .10 |  | 7.65 | .96 | .16 |  |
|  | **Pea** | 10.62 | .32 | .12 |  | 11.22 | .24 | .07 |  | 11.54 | .39 | .11 |  | 12.58 | .39 | .09 |  | 12.65 | .48 | .12 |  |
|  | **Lettuce** | 5.26 | .61 | .12 |  | 5.58 | .40 | .07 |  | 5.73 | .37 | .06 |  | 6.00 | .29 | .05 |  | 6.22 | .37 | .06 |  |
| **Dry biomass** | **Chilli** | .06 | .02 | .01 | .00 | .07 | .03 | .01 | .00 | .14 | .03 | .01 | .00 | .20 | .03 | .00 | .00 | .21 | .03 | .01 | .00 |
|  | **Tomato** | .08 | .03 | .00 |  | .11 | .02 | .00 |  | .13 | .02 | .01 |  | .16 | .03 | .005 |  | .19 | .03 | .006 |  |
|  | **Pea** | 1.52 | .19 | .06 |  | 1.68 | .18 | .06 |  | 1.98 | .23 | .07 |  | 2.02 | .26 | .07 |  | 2.21 | .22 | .06 |  |
|  | **Lettuce** | .06 | .03 | .004 |  | .06 | .02 | .004 |  | .15 | .04 | .006 |  | .23 | .06 | .01 |  | .25 | .12 | .02 |  |
